# Transcriptome Analysis for Abnormal Spike Development of the Wheat Mutant *dms*

**DOI:** 10.1371/journal.pone.0149287

**Published:** 2016-03-16

**Authors:** Xin-Xin Zhu, Qiao-Yun Li, Chun-Cai Shen, Zong-Biao Duan, Dong-Yan Yu, Ji-Shan Niu, Yong-Jing Ni, Yu-Mei Jiang

**Affiliations:** 1 National Centre of Engineering and Technological Research for Wheat, Henan Agricultural University / Key Laboratory of Physiological Ecology and Genetic Improvement of Food Crops in Henan Province, Zhengzhou, Henan, China; 2 Shangqiu Academy of Agricultural and Forestry Sciences, Shangqiu, Henan, China; Wuhan University, CHINA

## Abstract

**Background:**

Wheat (*Triticum aestivum* L.) spike development is the foundation for grain yield. We obtained a novel wheat mutant, *dms*, characterized as dwarf, multi-pistil and sterility. Although the genetic changes are not clear, the heredity of traits suggests that a recessive gene locus controls the two traits of multi-pistil and sterility in self-pollinating populations of the medium plants (M), such that the dwarf genotype (D) and tall genotype (T) in the progeny of the mutant are ideal lines for studies regarding wheat spike development. The objective of this study was to explore the molecular basis for spike abnormalities of dwarf genotype.

**Results:**

Four unigene libraries were assembled by sequencing the mRNAs of the super-bulked differentiating spikes and stem tips of the D and T plants. Using integrative analysis, we identified 419 genes highly expressed in spikes, including nine typical homeotic genes of the MADS-box family and the genes *TaAP2*, *TaFL* and *TaDL*. We also identified 143 genes that were significantly different between young spikes of T and D, and 26 genes that were putatively involved in spike differentiation. The result showed that the expression levels of *TaAP1-2*, *TaAP2*, and other genes involved in the majority of biological processes such as transcription, translation, cell division, photosynthesis, carbohydrate transport and metabolism, and energy production and conversion were significantly lower in D than in T.

**Conclusions:**

We identified a set of genes related to wheat floral organ differentiation, including typical homeotic genes. Our results showed that the major causal factors resulting in the spike abnormalities of *dms* were the lower expression homeotic genes, hormonal imbalance, repressed biological processes, and deficiency of construction materials and energy. We performed a series of studies on the homeotic genes, however the other three causal factors for spike abnormal phenotype of *dms* need further study.

## Introduction

Wheat (*Triticum aestivum* L.) floral organs provide the basis for grain formation such that wheat yield and quality are directly influenced by floral organ development [1]. Wheat spike abnormalities impact on wheat production, but these mutants are of great benefit to researches of genetics and breeding. Two types of abnormal pistil (gynoecia, ovary or carpel in different reports) mutants in wheat have been reported, multi-pistil and pistillody [2–5]. Most multi-pistil wheats belong to ‘tri-grain’ wheat, whose florets have a lemma, a palea, two lodicules, three stamens, and three pistils. Multi-pistil wheat is different from pistillody wheat, whose floral organ components have a lemma, a palea, two lodicules, a pistil and three pistil-like structures instead of stamens. The additional pistils of multi-pistil mutants are fertile, but those of pistillody mutants are sterile.

Genetic analyses indicate that the ‘tri-grain’ traits in various germplasms are controlled by either a single dominant gene [3, 4, 6, 7], a recessive gene [8], or two recessive noncomplementary genes [9, 10], and different cytoplasms [11] or alien cytoplasms as of *Aegilops kotschyi* and *Aegilops ventricosa* inhibit the expression of some ‘tri-grain’ genes [8]. The ‘tri-grain’ genes are located on 2DL, 5DS, 6BS and 6B respectively [7–10, 12]. The process of floral organ development in ‘tri-grain’ wheat is similar to that in cultivated common wheat, but the two additional pistils initiate after emergence of the first one [13–15]. Although ‘tri-grain’ wheat had been reported for several decades, the molecular mechanism of their floral organ development remains unknown.

Current studies indicate that abnormalities of crop spikes may be caused by a single gene, nuclear gene interraction, non-coding gene (ncRNA) [16], nuclei-cytoplasm interreaction [17–21] or microRNA regulation [22]. However, a mature spike is the consequence of a set gene expressions and interaction of complex gene regulatory networks (GRNs), rather than of any single gene expression, so changes of global gene expression profile in differentiating spikes are critical for wheat floral organ development studies. The whole transcriptome sequencing, combined with various developed bioligical databanks (SwissProt, TrEMBL, NR, NT, GO, KEGG etc; details see the part of materials and methods), provides a powerful technique for such researches [23].

Previously, we have obtained a dwarf, multi-pistil and sterility (*dms*) mutant from common wheat cultivar ‘Zhoumai 18’. The progeny of *dms* can be classified as dwarf (D), semidwarf (M) and tall (T) plants according to their plant height. D plants are dwarfism, multi-pistils and sterile. The heredity of the traits suggested that a recessive gene controlled the multi-pistil and sterility in self-pollination populations of M plants. The *dms* belongs to ‘multi-pistil’ mutant, but the plants of D have 1 ~ 6 pistils in one floret, that is different from ‘tri-grain’ wheats reported early. Furthermore, the plants of D have no seed setting at all (completely sterile) [24]. The availability of a set of germplasms of D, M and T genotypes provided an opportunity to design experiments that determine the gene expression profiles while floral organ differentiation. In this study, we reported the different expression profiles of D and T at early stage of spike specification, and assessed their potential roles in wheat floral organ specification.

## Materials and Methods

### Plant materials and growth conditions

**Fig 1:**
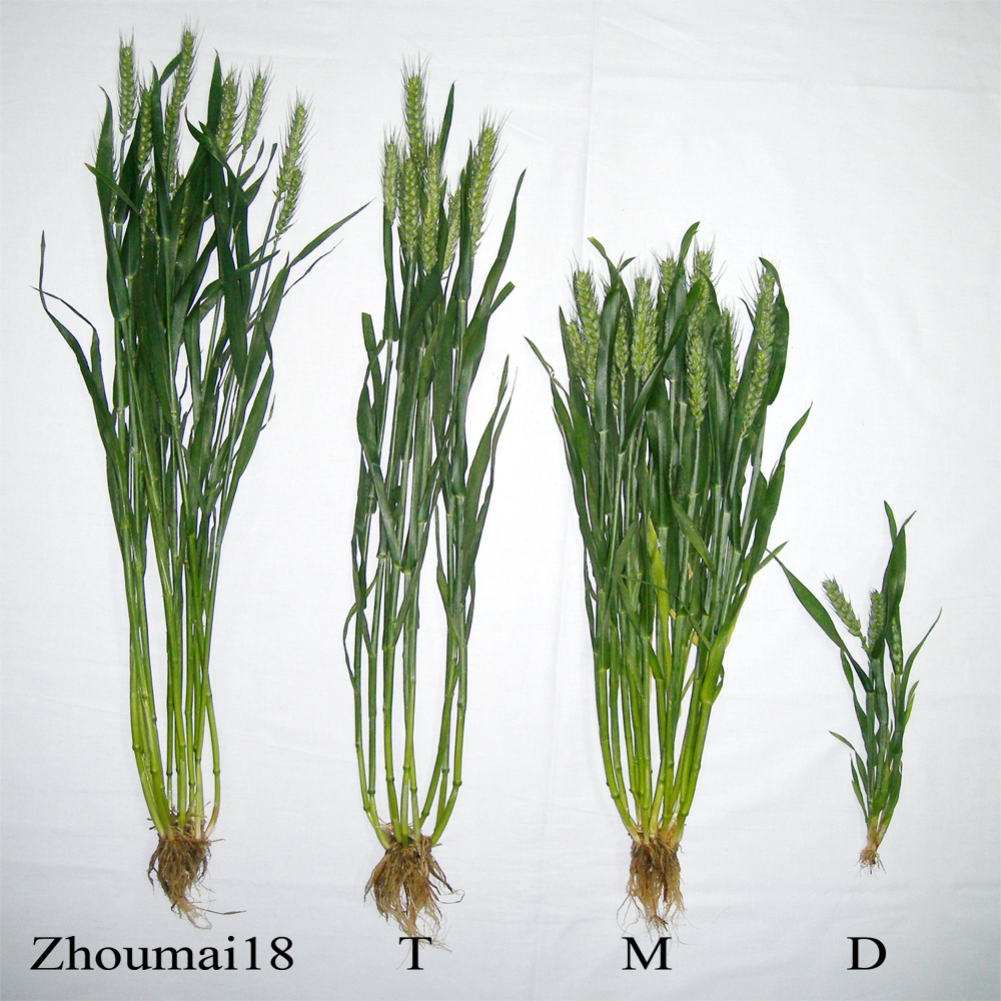
Individual plant morphology of ‘Zhoumai 18’ and *dms* mutant. ‘Zhoumai 18’: the parent plants of *dms* mutant, T: tall plants, M: semi-dwarf plants, D: dwarf plants.

Zhoukou Academy of Agricultural Sciences and Henan Agriculture University specifically permitted our field experiments. ‘Zhoumai 18’ was bred by Zhoukou Academy of Agricultural Sciences, Zhoukou, Henan, China. The *dms* mutant was derived from ‘Zhoumai 18’. The T, M and D in progeny of *dms* were selected by us on experimental field issued by Henan Agriculture University, Zhengzhou, Henan, China and conserved in our laboratory (Fig 1). The other traits such as tiller number, internode number, floret structure and fertility of both the semi-dwarf and tall plants were almost the same as that of ‘Zhoumai 18’, However, the dwarf plants (D) were significantly different from ‘Zhoumai 18’ and T plants, The average plant height, tiller number, and spike number per plant of D were only 0.29 m, 3.9 and 2.4, respectively, they decreased by 62.4, 80.5 and 84.6% compared with those of ‘Zhoumai 18’ and their spikes were sterile (Table 1) [25]. Individual plant lines of mutant *dms* were sown from 2009–2011. Because D plants do not produce seeds, the mutant gene was kept by sowing seeds from the M plants every year. The field experiments were carried out in a completely randomized design. Forty populations (eleven and 29 derived from the T and M plants of *dms* respectively) were sown in Henan Agriculture University experimental field in 2012–2013. The segregating populations were sown in plots of 3.0 m in length and 2 m in width, the distance between rows was 21 cm, 30 seeds were planted in each row. A hundred and twenty seeds of each population (the progeny of M, that segregated into T, M, and D at ratio of 1:2:1; the progeny of T that did not segregate any more) were sown. Fertilizer and weed management were similar to wheat breeding [25].

**Table 1:** Characteristics of genotypes in the 26 segregating populations in 2012–2013.

Traits *	T	M	D	Zhoumai 18
Plant height (cm)	76.2 ± 2.1^b^	57.3 ± 1.3^c^	29.5 ± 1.1^d^	78.5 ± 1.0^a^
Spike length (cm)	9.7 ± 0.7^ab^	9.3 ± 0.2^b^	7.1 ± 0.5^c^	10.2 ± 1.2^a^
Internode length on the main stem (cm)				
The first	5.6 ± 0.8^a^	2.0 ± 0.7^b^	-	6.2 ± 1.1^a^
The second	7.6 ± 0.4^a^	5.2 ± 0.3^b^	-	7.6 ± 0.3^a^
The third	10.1 ± 0.6^a^	7.3 ± 0.5^b^	3.0 ± 0.2^c^	10.1 ± 0.2^a^
The fourth	17.8 ± 0.7^a^	13.6 ± 0.7^b^	7.2 ± 0.2^c^	17.8 ± 0.4^a^
The fifth	25.3 ± 0.9^b^	20.0 ± 0.9^c^	12.2 ± 0.3^d^	26.7 ± 1.5^a^
Internode number of main stem	5.0 ± 0.0^a^	4.8 ± 0.2^b^	3.0 ± 0.0^c^	5.0 ± 0.0^a^
Tiller number	18.9 ± 2.2^ab^	18.2 ± 1.6^b^	3.9 ± 0.8^c^	20.0 ± 3.1^a^
Spike number	15.9 ± 1.6^a^	14.5 ± 1.6^a^	2.4 ± 0.7^b^	15.6 ± 2.5^a^
Spikelet number on the main stem	19.0 ± 0.6^a^	17.6 ± 0.6^b^	5.3 ± 1.7^c^	19.0 ± 0.0^a^
Seed number per spikelet	3–4	3–4	<3	3–4
Stamen number of floret	3	3	3	3
Pistil number of floret	1	1	1–6	1

* Means within a row followed by a different letter differ significantly according to **Duncan’s Multiple Range test at *p < 0*.*01*.**

There are only three internodes on the dwarf plants, the values are shown as the 3rd, 4th and 5th compared with those of the other genotypes [25].

### Observation of spikes and stem tips

**Fig 2:**
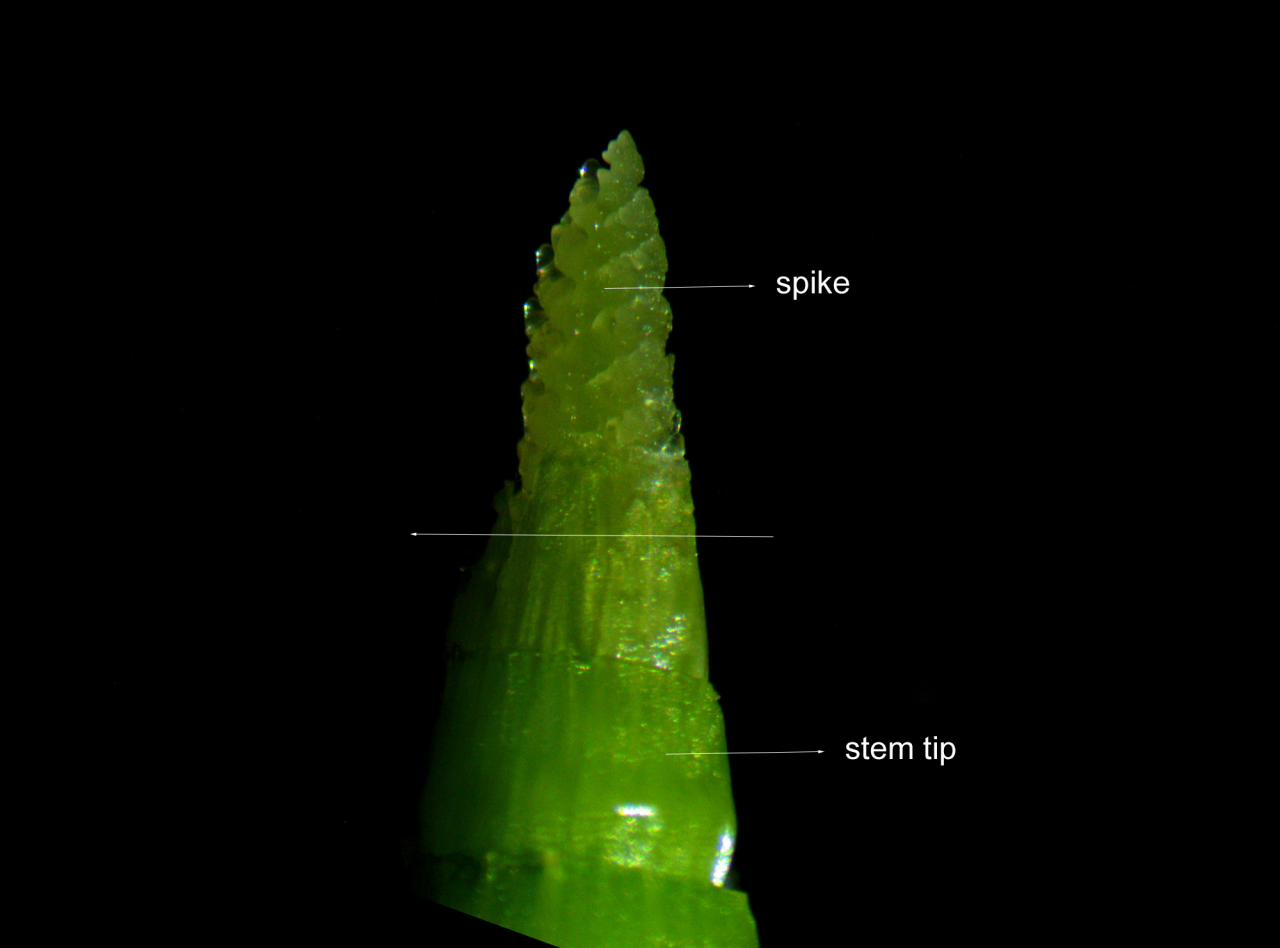
Sample of spike and stem tip.

The young spikes and young stem tips were observed with an inverted microscope (Olympus 3111286), and images were captured by a camera (Nikon Coolpix 4500). The young spikes at stages from glume to stamen / pistil primordium differentiation and young stem tips (small fragment just connected with young spikes) of D and T genotypes were taken from the segregating populations (progeny of M) (Fig 2). Each of the four super bulks was consisted of 20 individuals to average gene expression background and biological variability. Young spikes of D, M and T at different developmental stages [26] were taken for real time quantitative RT-PCR.

### Transcriptome mRNA sequencing

The transcriptome mRNA sequencing and analysis were carried out in BioMarker Company (Beijing, China). Total RNA was extracted using TRIzol reagent (Invitrogen, Carlsbad, CA, USA). Four super bulk samples: T1, young stem tips of D at Z31 stage (from 14 to 20 March 2013); T2, young spikes of D in the phase from glume to stamen / pistil primordium differentiation (from 15 to 22 March 2013); T3, young stem tips of T at Z31 stage (from 9 to 15 March 2013) and T4, young spikes of D in the phase from glume to stamen / pistil primordium differentiation (from 9 to 15 March 2013). Pair-end sequencing was carried out on Illumina HiSeq^TM^ 2000 using the standard protocol provided by Illumina company (San diego, CA, USA).

### Sequencing data analysis

The sequencing data were assembled and analyzed as no reference genome sequences. Transcriptomes (including contigs, transcripts and unigenes) of the four bulk samples were assembled using software Trinity [27] with parameters of K-mer = 25, group pairs distance = 500 (maximum length expected between fragment pair). Unigenes were clustered using Blat (The BLAST-Like Alignment Tool; http://genome.ucsc.edu/cgi-bin/hgBlat) with parameters of tile size = 8, step size = 5. Open reading frames (ORFs) of the unigenes in the assembled unigene library were analyzed using a program Getorf in a software pack EMBOSS (http://emboss.sourceforge.net/apps/cvs/emboss/apps/getorf.html). Sequence data of each sample were compared with gene sequences in unigene library, gene expression enrichment was analyzed based on the different numbers of reads. Quantitative gene expression was calculated with the method of RPKM [28]. The formula is:

RPKM=TotalExonReadsMappedReads(Millions)×ExonLength(KB)

Differentially expressed genes (DEGs) were searched based on gene expression enrichments in different samples using software IDEG6 (http://telethon.bio.unipd.it/bioinfo/IDEG6/), *p* < 0.01, ratio of RPKM ≥ 2 (Fold change ≥ 2). Cluster of DEGs was done using Cluster 3.0 with parameters of ca = m (specifies whether to center each row (gene) in the data; m: subtract the median of each row), g = 7 (specifies the distance measure for gene clustering; 7: euclidean distance), e = 7 (specifies the distance measure for microarray clustering; 7: euclidean distance). Gene annotation were carried out referred to several Annotation-Databases of NR and NT (http://www.ncbi.nlm.nih.gov/), SwissProt and TrEMBL (http://www.uniprot.org/), GO (http://www.geneontology.org/), COG (http://www.ncbi.nlm.nih.gov/COG/) and KEGG (http://www.genome.jp/kegg/). Significant enrichment analyses of GO and KEGG pathway for DEGs were carried out at the websites with corrected *p* ≤ 0.05.

### Real time QRT-PCR

To study the gene expression profiles in the whole spike developmental stage, total 64 samples of each genotype were prepared (S1 Table). Total RNA was extracted as described for sequencing. cDNA was synthesized from 1.5 μg of DNase-treated total RNA with 200 U M-MLV Reverse Transcriptase (Promega, Madison, USA) and oligo-dT primers. Real time QRT-PCR was performed using Go Taq® qPCR Master Mix (Promega, Madison, USA) according to the manufacturer’s protocol on the CFX Connect^TM^ Real-Time System (Bio-Rad, Hercules, CA, USA). Wheat *Actin* and *gadph* genes were used as internal controls for normalization. The gene specific primers were listed in S2 Table.

## Results

### The expressions of most genes in differentiating spikes of D were lower

**Fig 3:**
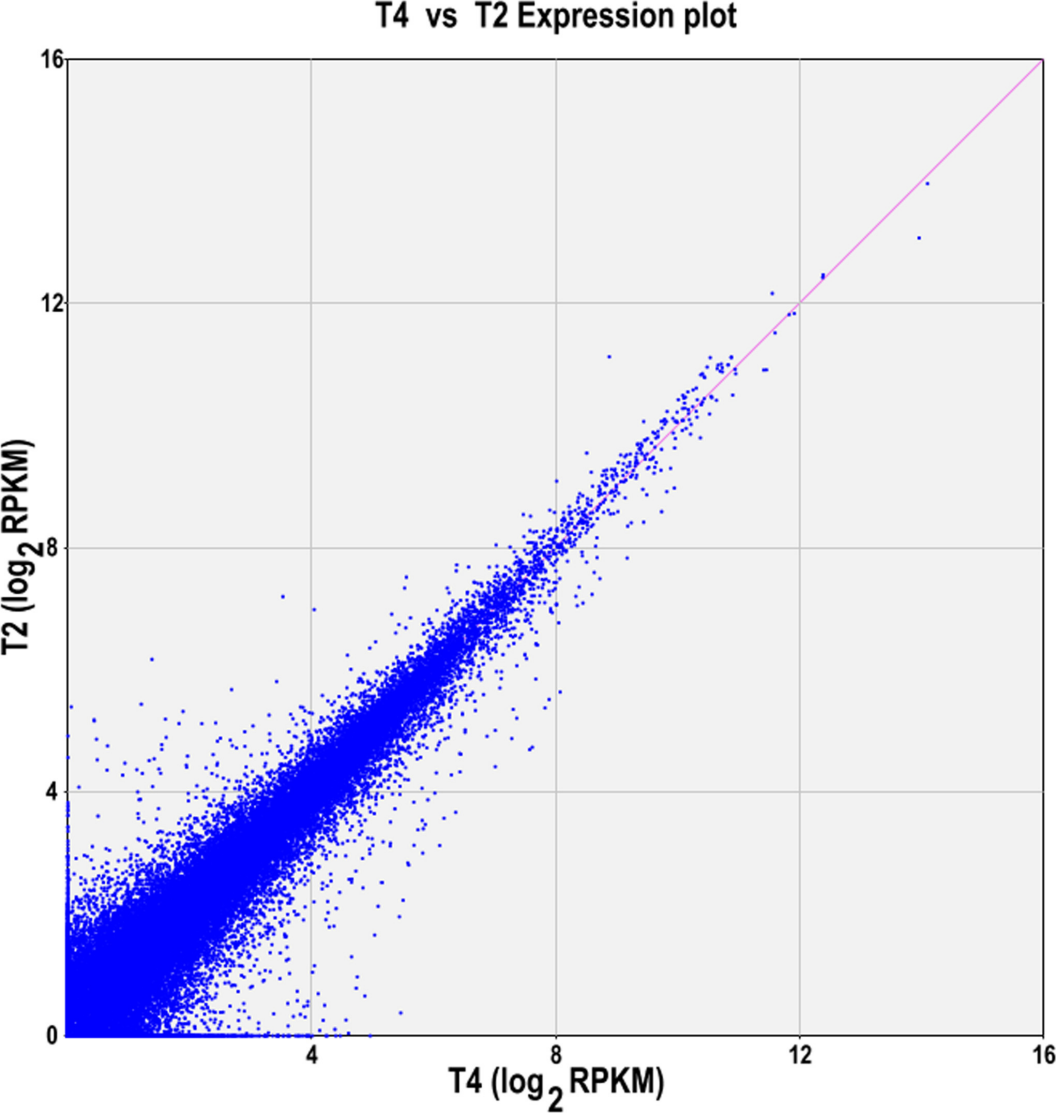
Scatterplot of gene expression level in differentiating spikes of T (T4) and D (T2). One dot indicates one gene, the farther from the diagonal line the higher gene expression level was. RPKM, reads per kilobase per million reads.

Through whole transcriptome mRNA sequencing, a total 27.59 G data, 136,596,533 pair reads were obtained from the four super bulk samples: young stem tips of D, young spikes of D, young stem tips of T and young spikes of T (sample number: T1, T2, T3 and T4). Total 128,619 unigenes were *de novo* assembled, 20,296 of them were over 1 kb in length. 90,424 unigenes were annotated for biological functions. The transcriptome data of the young spikes on T and D were similar, including reads number (35,703,321; 35,329,399), base number (7,211,384,222; 7,135,855,487) and *de novo* assembled unigenes (65,316; 63,800). Generally, the gene expression levels of differentiating spikes of T were relatively higher than that of D (Fig 3).

### The genes involved in wheat floral organ specification

**Fig 4:**
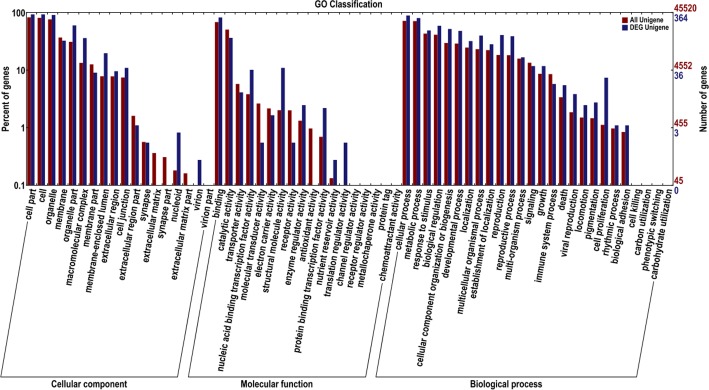
Functional classification of the differentially expressed genes (DEGs) that highly expressed in differentiating spikes of both T and D.

To identify the genes involved in wheat floral organ specification, the transcriptomes of young spikes on D and T were compared with those of their stem tips. Total 419 genes highly expressed in spikes had been screened out. Functional classification referred to GO showed that the following aspects were significantly different between wheat differentiating spikes and their stem tips: (1) cellular component: organelle part, macromolecular complex, membrane-enclosed lumen, cell junction and nucleoid; (2) molecular function: nucleic acid binding transcription factor, molecular transducer, structural molecule, receptor, enzyme regulator, protein binding transcription factor and transporter activity; (3) biological process: biological regulation, metabolic process, cellular component organization or biogenesis, developmental process, locomotion, multicellular organismal process, establishment of localization, reproductive process, reproduction, death and cell proliferation (Fig 4). Except for molecular transducer, receptor and antioxidant activity, all the others were more active in differentiating spikes than in their stem tips. These suggested that the most gene expressions, cell structure and major biological processes were activated when transition from vegetative to reproductive growth. The active biological processes of biological regulation, developmental process, reproductive process and cell proliferation exactly reflected the typical characters of the differentiating spikes, which were at the early stage of wheat spike specification.

The KEGG analysis suggested that the purine and pyrimidine metabolism, DNA replication (S1 Fig), nucleotide excision repair, base excision repair, mismatch repair, spliceosome (S2 Fig), ribosome biogenesis (S3 Fig) were more active in differentiating spikes than in their stem tips. The data suggested that the gene expression process played a crucial role at the early stage of spike specification (Table 2).

**Table 2:** Compared with their stem tips, the up-regulated pathways in wheat spikes at early stage of floral organ differentiation.

Biological process	Up-regulated Pathway	Ko ID*	Unigene with pathway annotation	gene with pathway annotation
**Replication**	DNA replication	ko03030	15	67
**(50 genes)**	Mismatch repair	ko03430	7	52
	Homologous recombination	ko03440	4	63
	Nucleotide excision repair	ko03420	7	89
	Base excision repair	ko03410	7	63
	Purine metabolism	ko00230	4	213
	Pyrimidine metabolism	ko00240	5	180
	Non-homologous end-joining	ko03450	1	19
**Transciption**	Spliceosome	ko03040	12	287
**(19 genes)**	Basal transcription factors	ko03022	1	63
	mRNA surveillance pathway	ko03015	2	252
	RNA transport	ko03013	1	345
	RNA degradation	ko03018	3	131
**Translation**	Ribosome	ko03010	24	332
**(41 genes)**	Ribosome biogenesis in eukaryotes	ko03008	6	154
	Cysteine and methionine metabolism	ko00270	3	124
	Arginine and proline metabolism	ko00330	3	97
	Glutathione metabolism	ko00480	1	81
	Protein processing in endoplasmic reticulum	ko04141	1	218
	Ubiquitin mediated proteolysis	ko04120	3	165
**Carbohydrate metabolism**	Amino sugar and nucleotide sugar metabolism	ko00520	1	123
	Starch and sucrose metabolism	ko00500	1	185
**Other metabolism**	ABC transporters	ko02010	1	16
	Inositol phosphate metabolism	ko00562	1	81
	Phagosome	ko04145	1	110
	Natural killer cell mediated cytotoxicity	ko04650	1	25

Note: All unigene: 80; All gene: 5389.

* Number of the metabolism pathway in Kanehisa Laboratories.

According to gene functional annotation, 40 genes among the 419 genes highly expressed in differentiating spikes were putatively involved in wheat spike development (Table 3). The genes of DNA replication, transcription, translation factors (gene expression), ABC transporter B family, protein kinase, carbohydrate metabolism, cytochrome, signal transduction and general function prediction only were involved in the development of various components of wheat floral organ (Table 3). Both ‘gene expression’ and ‘carbohydrate metabolism’ had nine unigenes each (total 18 genes) that implied their important roles in spike differentiating. The genes of ABC transporter B family might also played an important role because three significant DEGs had been found, that suggested transmembrane production transport was key for floral organ differentiation.

**Table 3:** The DEGs involved in wheat floral organ differentiation.

Unigene ID	Gene ID**, annotation, PE value, SV value (Swissprot or TrEMBL database)	Functional annotation (GO) ***
	**Homeotic genes**	
**T4-56460**	sp|Q6EU39|; MADS-box transcription factor 6 OS = O. sativa subsp. japonica GN = MADS6 PE = 1 SV = 1	1, 2
**T1-42731**	sp|Q6Q9I2|; MADS-box transcription factor 15 OS = O. sativa subsp. japonica GN = MADS15 PE = 1 SV = 2	3, 4, 5
**T4-52821**	sp|Q6Q9I2|; MADS-box transcription factor 15 OS = O. sativa subsp. japonica GN = MADS15 PE = 1 SV = 2	3, 4, 5
**T3-69731**	sp|Q8S151|; MADS-box transcription factor 32 OS = O. sativa subsp. japonica GN = MADS32 PE = 2 SV = 1	4, 6, 7
**T2-44069**	sp|Q6Q9H6|; MADS-box transcription factor 34 OS = O. sativa subsp. japonica GN = MADS34 PE = 2 SV = 2	8, 1, 9, 10, 6, 2
T4-56463*	sp|Q0D4T4|; MADS-box transcription factor 18 OS = O. sativa subsp. japonica GN = MADS18 PE = 1 SV = 1	
T3-3226*	sp|Q69TG5|; MADS-box transcription factor 55 OS = O. sativa subsp. japonica GN = MADS55 PE = 2 SV = 2	
T3-8854*	sp|Q5K4R0|; MADS-box transcription factor 47 OS = O. sativa subsp. japonica GN = MADS47 PE = 1 SV = 2	
**T1-71336**	sp|Q38914|; AP2-like ethylene-responsive transcription factor ANT OS = A. thaliana GN = ANT PE = 1 SV = 2	11, 1, 6
**T2-61506**	sp|Q38914|; AP2-like ethylene-responsive transcription factor ANT OS = A. thaliana GN = ANT PE = 1 SV = 2	11,1
T2-51524*	sp|Q9FH95|; AP2-like ethylene-responsive transcription factor TOE3 OS = A. thaliana GN = TOE3 PE = 2 SV = 1	
**T4-56456**	sp|A2XX39|; ranscription factor FLORICAULA/LFAFY (FL) OS = O. sativa subsp. indica GN = OSL PE = 2 SV = 2;	4, 5
**T4-65297**	sp|Q76EJ0|; Protein DROOPING LEAF (DL) OS = O. sativa subsp. japonica GN = DL PE = 1 SV = 1	8, 1, 12, 14, 13, 2
	**DNA replication, transcription, translation factors (‘gene expression’)**	
**T4-55714**	sp|Q8VYZ0|; E3 ubiquitin-protein ligase ORTHRUS 2 OS = A. thaliana GN = ORTH2 PE = 1 SV = 1	21, 17
**T2-59388**	sp|B1Q3J6|; DNA (cytosine-5)-methyltransferase 1B OS = O. sativa subsp. japonica GN = MET1B PE = 2 SV = 1	29
**T2-60647**	sp|Q0DGS1|; Auxin response factor 14 OS = O. sativa subsp. japonica GN = ARF14 PE = 2 SV = 2	16, 22
**T2-61031**	sp|Q9M2U1|; Dof zinc finger protein DOF3.6 OS = A. thaliana GN = DOF3.6 PE = 1 SV = 2	23
**T2-47395**	sp|Q8S857|; Probable histone H2A variant 2 OS = O. sativa subsp. japonica GN = Os10g0418000 PE = 2 SV = 1	8, 24
**T4-61443**	sp|Q9P281|; BAH and coiled-coil domain-containing protein 1 OS = Homo sapiens GN = BAHCC1 PE = 1 SV = 3	28
**T1-36780**	sp|Q7Y0V9|; Homeobox-leucine zipper protein ROC4 OS = O. sativa subsp. japonica GN = ROC4 PE = 2 SV = 2	25, 26
**T4-16753**	sp|Q8VZT0|; Putative H/ACA ribonucleoprotein complex subunit 1-like protein 1 OS = A. thaliana GN = At3g03920 PE = 2 SV = 1	8
**T3-65265**	sp|Q9LEY9|; H/ACA ribonucleoprotein complex subunit 2-like protein OS = A. thaliana GN = At5g08180 PE = 1 SV = 1	8
	**ABC transporter B family**	
**T3-68332**	sp|Q9LJX0|; ABC transporter B family member 19 OS = A. thaliana GN = ABCB19 PE = 1 SV = 1	17, 13, 10
**T3-18532**	sp|Q9LJX0|; ABC transporter B family member 19 OS = A. thaliana GN = ABCB19 PE = 1 SV = 1	17, 13, 10
**T3-18860**	sp|Q9LJX0|; ABC transporter B family member 19 OS = A. thaliana GN = ABCB19 PE = 1 SV = 1	13, 10
	**Protein kinase**	
**T4-16606**	sp|Q42371|; LRR receptor-like serine/threonine-protein kinase ERECTA OS = A. thaliana GN = ERECTA PE = 1 SV = 1	18, 8, 19, 14, 9, 6, 20
**T4-54940**	sp|Q9C9N5|; Probable inactive LRR receptor-like protein kinase At1g66830 OS = A. thaliana GN = At1g66830 PE = 1 SV = 1	16
	**Carbohydrate metabolism**	
**T3-9854**	sp|Q6Z2J3|; Endoglucanase 6 OS = O. sativa subsp. japonica GN = Os02g0733300 PE = 2 SV = 1	1, 13
**T1-41804**	sp|Q8LQ92|; Endoglucanase 3 OS = O. sativa subsp. japonica GN = GLU8 PE = 2 SV = 1	1, 13
**T2-57113**	sp|Q8LQ92|; Endoglucanase 3 OS = O. sativa subsp. japonica GN = GLU8 PE = 2 SV = 1	1, 13
**T4-38224**	sp|Q8LQ92|; Endoglucanase 3 OS = O. sativa subsp. japonica GN = GLU8 PE = 2 SV = 1	1, 13
**T4-60868**	sp|Q652F9|; Endoglucanase 17 OS = O. sativa subsp. japonica GN = GLU13 PE = 2 SV = 1	1
**T4-16167**	sp|Q05091|; Polygalacturonase inhibitor OS = Pyrus communis GN = PGIP PE = 1 SV = 1	25, 26
**T2-18461**	sp|P86857|; Alanine and glycine-rich protein (Fragment) OS = Mytilus californianus PE = 1 SV = 1	16
**T2-57117**	sp|Q8LQ92|; Endoglucanase 3 OS = O. sativa subsp. japonica GN = GLU8 PE = 2 SV = 1	1, 13
**T3-9904**	sp|Q8LQ92|; Glucan endo-1,3-beta-glucosidase 13 OS = A. thaliana GN = At5g56590 PE = 1 SV = 1	8, 27, 24
	**Cytochrome**	
**T1-68702**	sp|O48927|; Cytochrome P450 78A3 OS = Glycine max GN = CYP78A3 PE = 2 SV = 1	15
**T2-60444**	sp|O48927|; Cytochrome P450 78A3 OS = Glycine max GN = CYP78A3 PE = 2 SV = 1	15
	**Signal transduction**	
**T2-44951**	sp|Q6P5M2|; WD repeat-containing protein 61 OS = Danio rerio GN = wdr61 PE = 2 SV = 1	29
	**General function prediction only**	
**T4-33022**	sp|Q8H1D3|; BTB/POZ domain-containing protein NPY1 OS = A. thaliana GN = NPY1 PE = 2 SV = 1	28, 15
**T3-10311**	sp|O22607|; WD-40 repeat-containing protein MSI4 OS = A. thaliana GN = MSI4 PE = 1 SV = 3	16,
**T3-67649**	tr|I1GRZ8|; Uncharacterized protein GN = BRADI1G20320 OS = B. distachyon (Purple false brome) PE = 4 SV = 1	8, 17, 15
**T1-61163**	sp|Q9SSD1|; Protein TOO MANY MOUTHS OS = A. thaliana GN = TMM PE = 2 SV = 1	16, 19, 10

* Genes additionally searched from DEGs of T1 vs T2 and T3 vs T4.

** The accession number with ‘sp’ is that of Swissprot database, with ‘tr’ is that of TrEMBL database. *A*. *thaliana*: *Arabidopsis thaliana*; *O*. *sativa*: *Oryza sativa*; *B*. *distachyon*: *Brachypodium distachyon*.

*** Annotation according to GO database.

1, specification of floral organ identity (GO:0010093)

2, specification of floral organ number (GO:0048833)

3, maintenance of floral meristem identity (GO:0010076)

4, maintenance of inflorescence meristem identity (GO:0010077)

5, floral meristem determinacy (GO:0010582)

6, ovule development (GO:0048481)

7, regulation of reproductive process (GO:2000241)

8, regulation of flower development (GO:0009909)

9, petal development (GO:0048441)

10, stamen development (GO:0048443)

11, regulation of floral meristem growth (GO:0010080)

12, inflorescence meristem growth (GO:0010450)

13, carpel development (GO:0048440)

14, seed development (GO:0048316)

15, inflorescence development (GO:0010229)

16, flower development (GO:0009908)

17, vegetative to reproductive phase transition of meristem (GO:0010228)

18, pollen tube growth (GO:0009860)

19, inflorescence morphogenesis (GO:0048281)

20, anther development (GO:0048653)

21, pollen development (GO:0009555)

22, vegetative phase change (GO:0010050)

23, floral organ abscission (GO:0010227)

24, floral organ formation (GO:0048449)

25, floral organ development (GO:0048437)

26, floral whorl development (GO:0048438)

27, pollen exine formation (GO:0010584)

28, positive regulation of flower development (GO:0009911)

29, negative regulation of flower development (GO:0009910).

**Fig 5:**
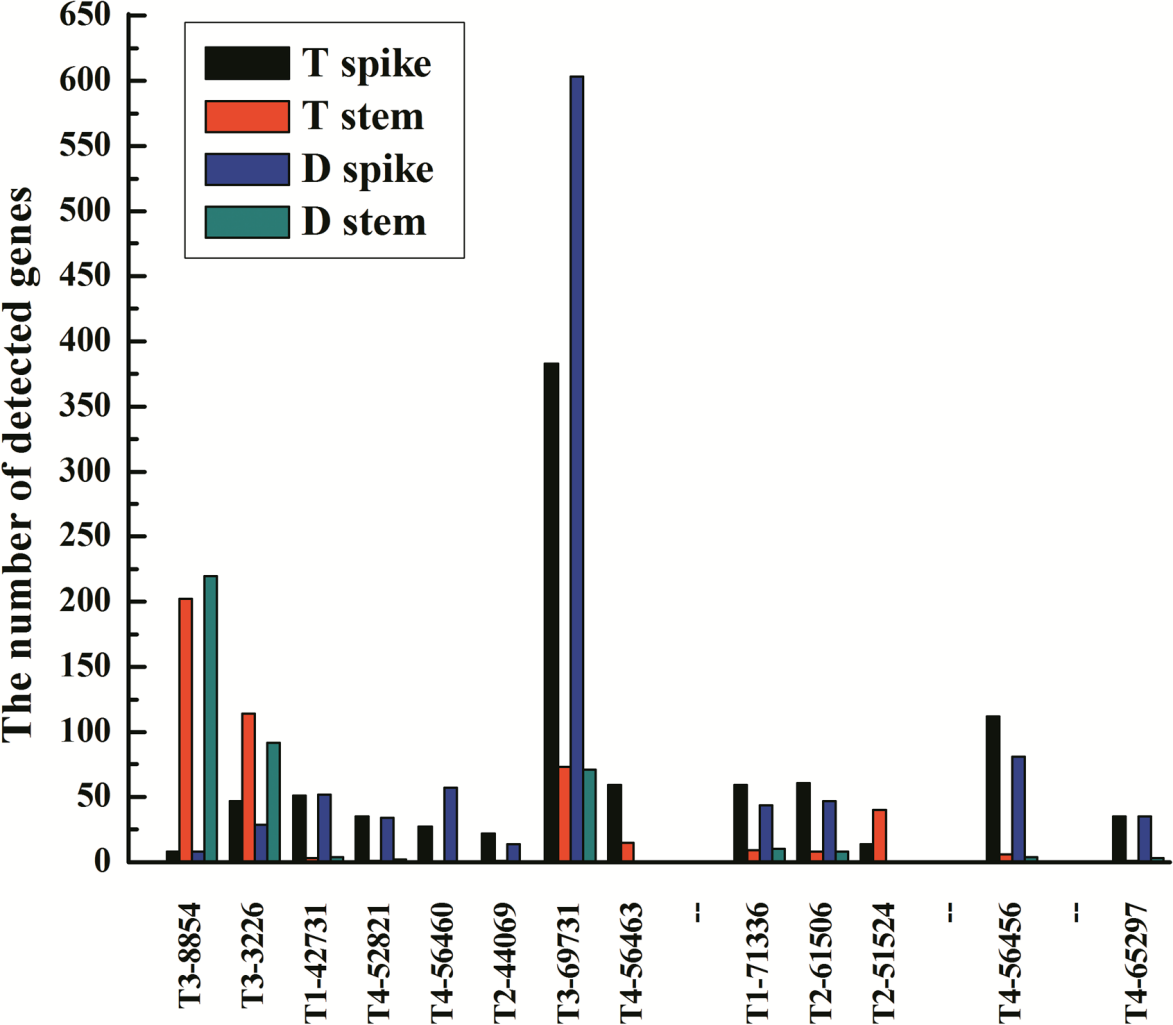
The transcriptional profile of the wheat homeotic genes. From left to right, the first eight unigenes are homologs of MADS-box family, the ninth to eleventh unigenes are *AP2-like* genes, the twelveth unigene (T4-56456) is *TaFL*, the thirteenth unigene (T4-65297) is *TaDL*. The homologs and annotation of the unigenes under the Fig are listed in Table 3.

Noticeably, nine of the 40 floral organ differentiation related genes belonged to the typical floral organ determining genes, homeotic genes, including MADS-box transcription factor, *AP2*-like ethylene-responsive transcription factor, *FLORICAULA*/*LEAFY* (*FL*) and *DROOPING LEAF* (*DL*) genes. To further explore the homeotic genes in the unigene libraries, we searched them in the DEGs of T1 vs T2 and T3 vs T4, four more homeotic genes were found (Table 3, marked with ‘*’). The homolog of *Oryza sativa MADS47* (*OsMADS47*) and homolog of *OsMADS55* were significantly highly expressed in stem tips than in differentiating spikes of both D and T; homolog of *OsMADS6* and homolog of *OsMADS34* almost only expressed in differentiating spikes. A homolog (T4-56463) of *OsMADS18* was highly expressed in differentiating spikes than in stem tips of T, contrarily, no copy was detected in D genotype. One more *AP2*-like ethylene-responsive transcription factor gene (T2-51524) was found in T, but none in D. The significantly high expression or exclusive expression in spikes suggested the homeotic genes played a determining role in wheat floral organ differentiation (Fig 5).

### The significant DEGs between spikes of T and D

**Fig 6:**
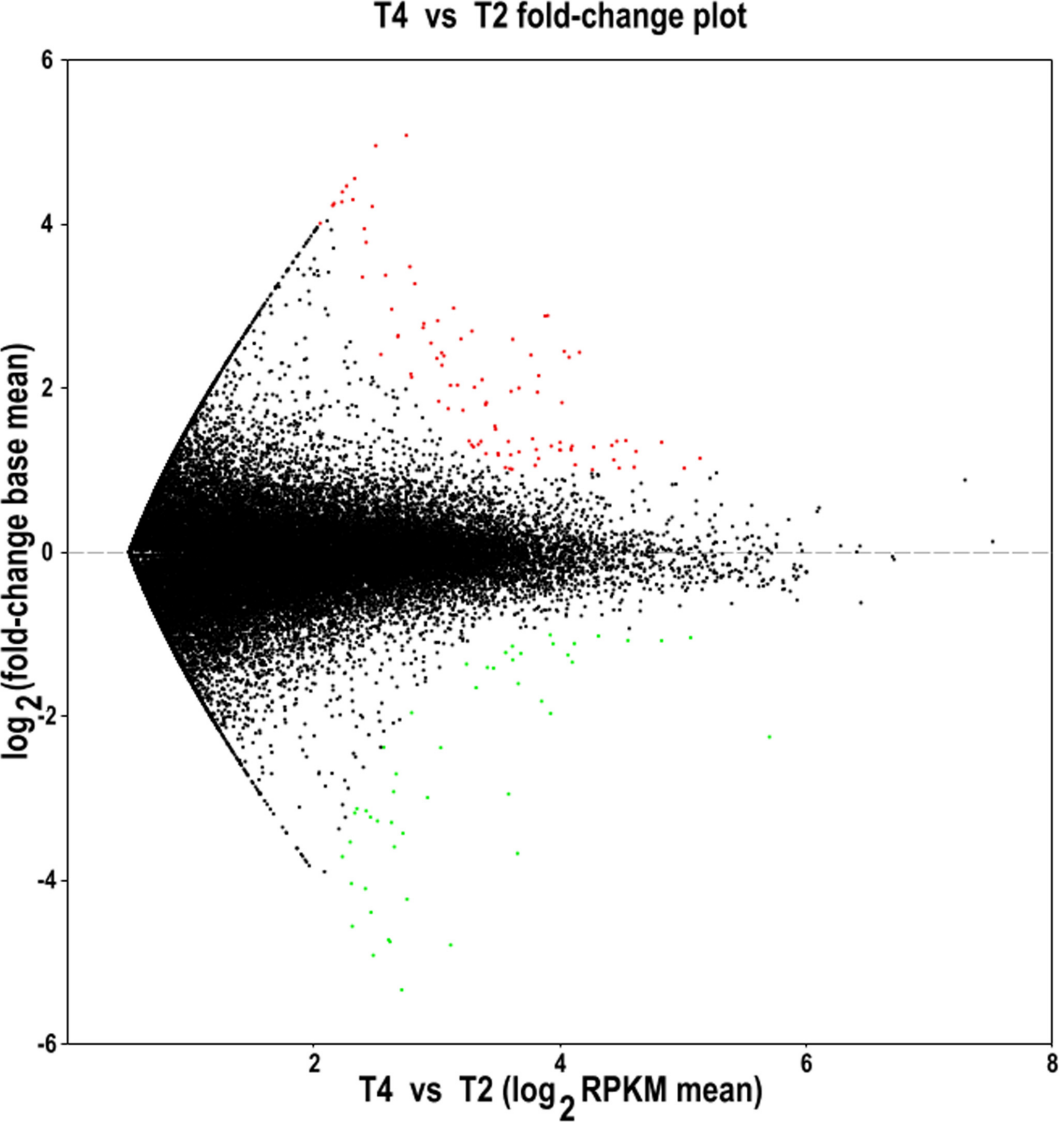
Fold-change plot of gene expression level in young spikes of T (T4) and D (T2). Black dot: gene expression changed insignificantly, red dot: significantly up-regulated gene, green dot: significantly down-regulated gene. RPKM, reads per kilobase per million reads.

The spikes of D are male sterile with 1–6 pistils in each floret, the other genotypes are normal and fertile. To explore the genes whose expressions were significantly different in spikes of T and D, the differentially expressed genes (DEGs) of differentiating spikes were carefully screened. A total 143 DEGs were obtained. Regarding D as control, 94 genes were significantly up regulated and 49 genes were down regulated in differentiating spikes of T (Fig 6; Table 4; S3 Table).

**Table 4:** Some important DEGs involved in spike differentiation.

Functional class	DEG ID	Log2 (T4/T2)	Gene ID**, annotation (SwissProt or TrEMBL annotation)	Putative function or biological processe (GO annotation)
**Cellular component**	T2-53412	-2.49	sp|Q6K602|; Protein Ruptured Pollen Grain 1	pollen development; anther dehiscence
	T4-20296*	2.35	sp|Q652A8|; UDP-glucose 4-epimerase 3	pollen development; root epidermal cell differentiation
	T2-39457	-3.7	sp|Q08000|; EM3_WHEAT, Em protein H2; Em-D1	regulation of flower development; vegetative to reproductive phase transition of meristem
**Energy production and conversion**	T3-37606	1.96	sp|A2YIW7|; Thioredoxin H-type	cell wall
**Replication, recombination and repair**	T4-58706*	4.58	sp|Q92372|; Replication factor A protein 1	Replication, recombination and repair
**Transcription**	T2-47003*	1.84	sp|Q94BN0|; BTB/POZ and TAZ domain-containing protein 2	pollen development; system development
	T2-47331*	-4.43	sp|Q5ZMJ9|; Serine/arginine repetitive matrix protein 1	RNA processing; nuclear part
	T4-42005	-3.58	sp|Q9T0K5|; Leucine-rich repeat extensin-like protein 3	developmental process involved in reproduction
	T1-64651	-3.09	sp|Q10QF2|; Homeobox-leucine zipper protein HOX12	root development
	T2-16245	14.36	sp|Q6ZQP7|; Uncharacterized protein LOC284861	RNA processing; transport; nuclear part
**Translation, ribosomal structure and biogenesis**	T3-10313	1.21	sp|Q945F4|; Eukaryotic translation initiation factor 5A-2	translation initiation factor activity
	T4-61340	1.07	sp|Q9FN11|; LOB domain-containing protein 37	simple leaf morphogenesis
	T2-41011*	-14.49	sp|Q9FR37|; Amidase 1	leaf morphogenesis; cell differentiation
**Posttranslational modification**	T1-38135*	1.38	sp|Q0J4P2|; Heat shock protein 81–1	flower development; leaf development
	T4-55787	-1.11	sp|P27484|; Glycine-rich protein 2	stamen development
	T1-59183	-2.08	sp|Q5Z5B2|; Protein argonaute 1D	flower morphogenesis
**Amino acid transport and metabolism**	T3-2970	2.1	sp|Q8GT75|; NEP1-interacting protein1	stamen development
	T2-50731	-14.87	sp|P31862|; Bowman-Birk type wound-induced proteinase inhibitor WIP1; wali5	serine-type endopeptidase inhibitor activity;
**Signal transduction mechanisms**	T1-59554	-2.98	sp|Q9SPK4|; Phosphoenolpyruvate carboxylase kinase 1	regulation of pollen tube growth
	T3-13958	-1.62	sp|Q39030|; Serine/threonine-protein kinase AtPK2/AtPK19	negative regulation of cell proliferation; positive regulation of cell growth
	T1-56016*	-5.17	sp|A2X7U1|; Homeobox-leucine zipper protein HOX24	transcription regulatory region sequence-specific DNA binding; regulation of transcription
**Response to out stimulus**	T4-918	4.7	sp|P14928|; cold acclimation protein WCOR615; ABA-inducible protein PHV A1	organic cyclic compound binding
	T3-66375	-1.32	sp|Q948Z4|; gibberellin stimulated transcript (GAST1)	cell proliferation
**Cell cycle control, cell division, chromosome partitioning**	T2-46647	1.35	sp|P42736|; Ethylene-responsive transcription factor RAP2-3	organ senescence
	T1-47968*	1.22	sp|O02414|; Dynein light chain LC6, flagellar outer arm	pollen tube growth
	T4-51695	1.27	sp|O02414|; Dynein light chain LC6, flagellar outer arm	pollen tube growth; regulation of cell proliferation
	T2-40114	1.29	sp|Q759T0|; Dynein light chain LC6, flagellar outer arm	root hair cell development
**Carbohydrate transport and metabolism**	T4-57047	3	sp|Q9SDL8|; Fructose-1,6-bisphosphatase, cytosolic	negative regulation of cell growth
	T4-52638*	14.14	sp|Q7XUR3|; Putative alpha-L-fucosidase 1	monooxygenase activity; oxidation-reduction process
	T1-71202*	-1.01	sp|Q0WUI9|; trehalose-6-phosphate synthase 2	specification of floral organ identity; carpel development; meristem development
	T3-5013	2.19	sp|Q2UXF7|; 6FEH_WHEAT, Fructan 6-exohydrolase	sucrose alpha-glucosidase activity; cell wall
**Inorganic ion transport and metabolism**	T2-43231	-3.27	sp|Q67YC0|; Inorganic pyrophosphatase 1	organ development
**General function**	T1-49256*	-5.36	sp|P20186|; Uncharacterized 35.5 kDa protein in Tn4556; LEA protein 12 (LEA12)	alcohol dehydrogenase (NAD) activity; response to salt stress; response to hypoxia; response to stress
	T2-35350	-3.73	sp|Q42980|; Oleosin 16 kDa	vegetative to reproductive phase transition of meristem; regulation of flower development
	T4-55876	1.36	sp|Q8BTI8|; Serine/arginine repetitive matrix protein 2	protein binding; vegetative phase change
**Unknown**	T2-14438*	4.86	tr|R7W5L8|; hypothetical protein F775_20975 (A. tauschii)	
	T3-33825	4.32	tr|F2CYY9|; Predicted protein	SNP on chromosome 3B

T2: gene nubmer of spikes on D, T4 gene number of spikes on T.

**Fig 7:**
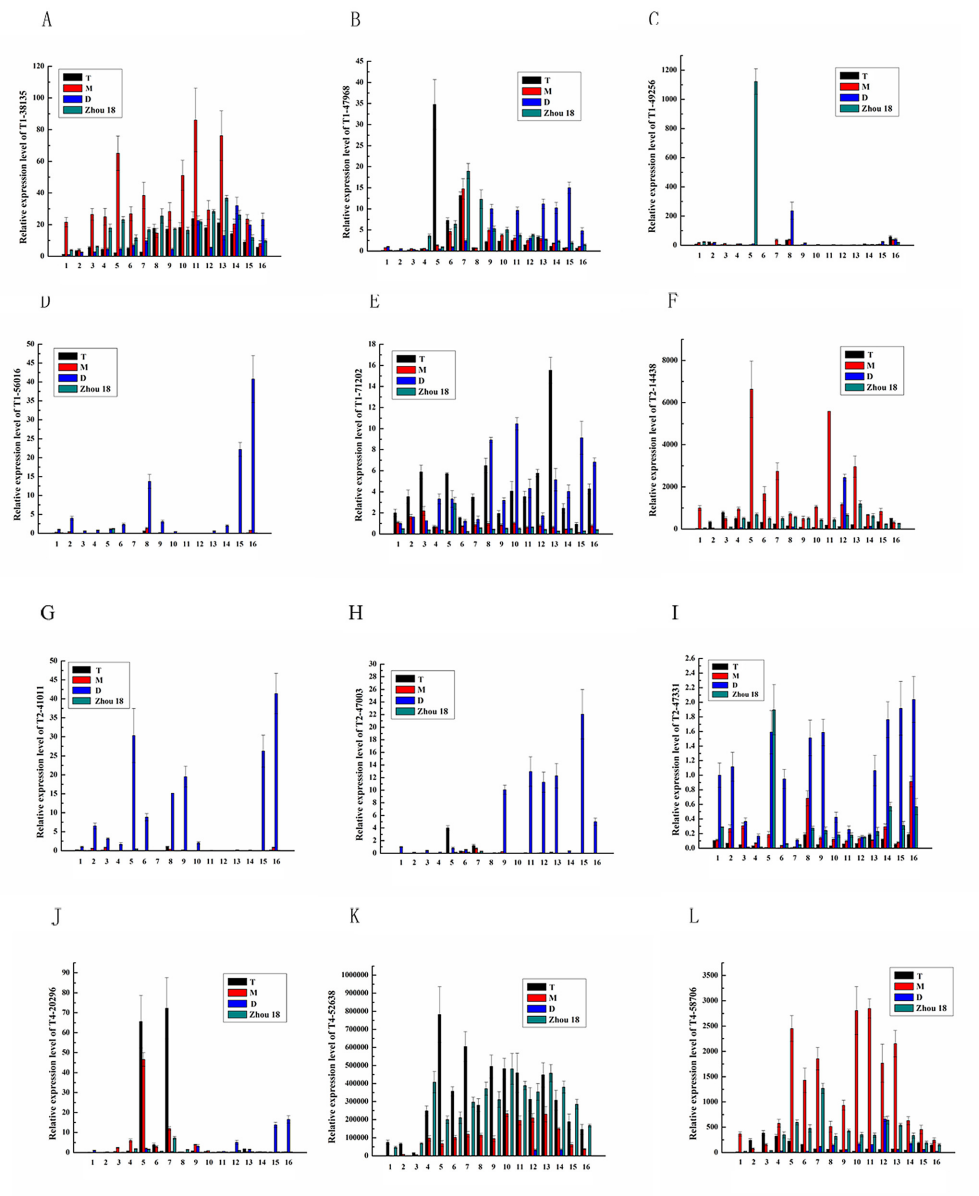
Temporal expression profiles of twelve genes in wheat differentiating spikes. A: unigene T1-38135; B: T1-47968 (*TaLC6*); C: T1-49256; D: T1-56016; E: T1-71202; F: T2-14438; G: T2-41011; H: T2-47003; I: T2-47331; J: T4-20296; K: T4-52638; L: T4-58706. Functional annotation of the unigenes (their homologs or similar genes) see Table 4. The *actin* gene was used as internal control. The number 1–16 indicate the sampling dates, and the details see S1 Table.

**Fig 8:**
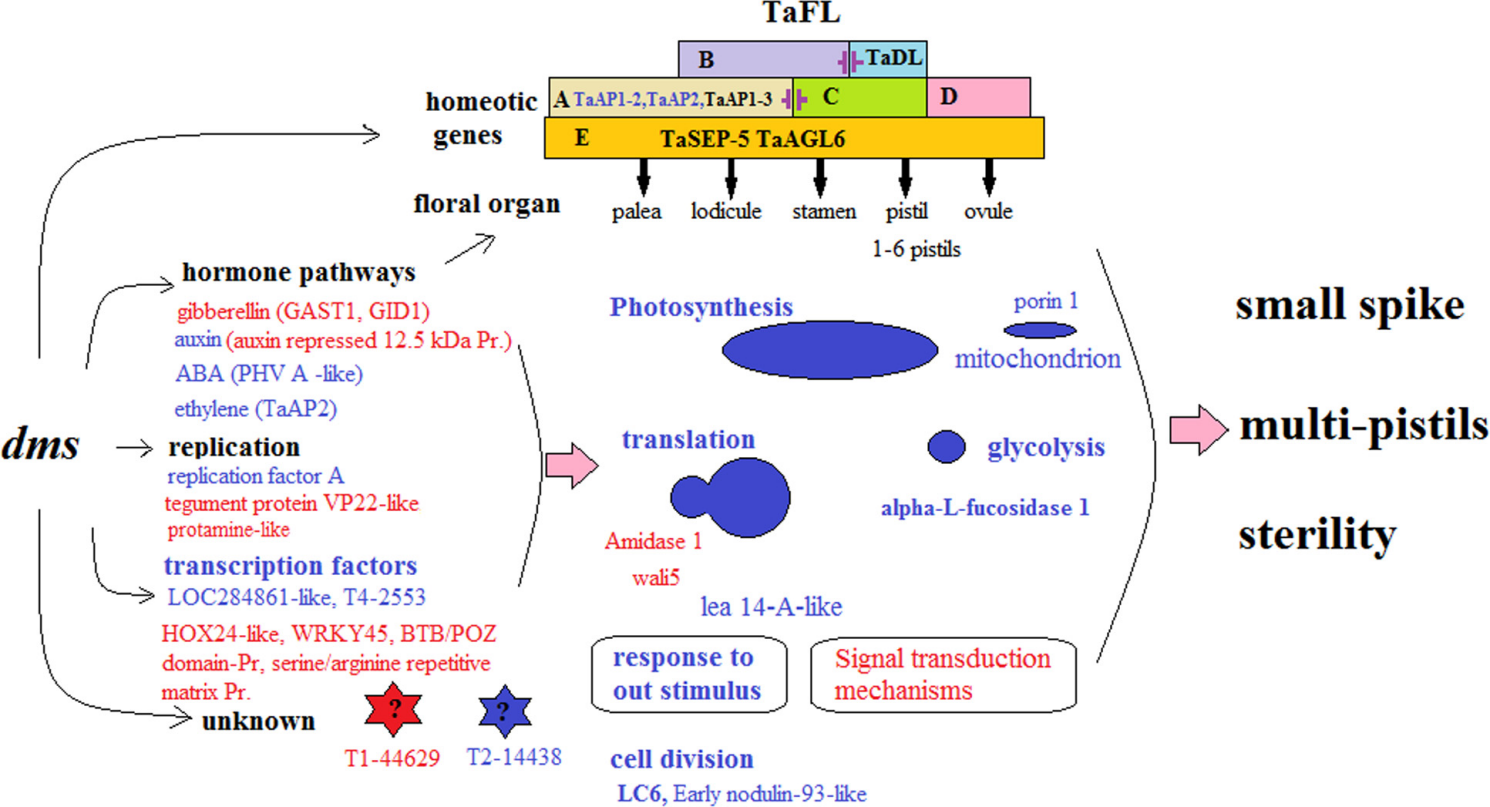
The transcriptome of D in differentiating spikes. The letters and Figs in red indicate significantly activated, in blue indicate significantly suppressed. The A, B, C, D and E in rectangles indicate different classes of homeotic genes. Class B and TaDL proteins show mutual suppression, which was suggested from analysis of a pistillody line. The mutual suppression between class A and C genes is also postulated [29]. Pr. is protein for short.

* Genes whose spatio-temporal expression profile was analyzed by real time QTR-PCR (Figs 7 and 8).

** The accession number with ‘sp’ is that of database SwissProt, with ‘tr’ is that of TrEMBL

**Fig 9:**
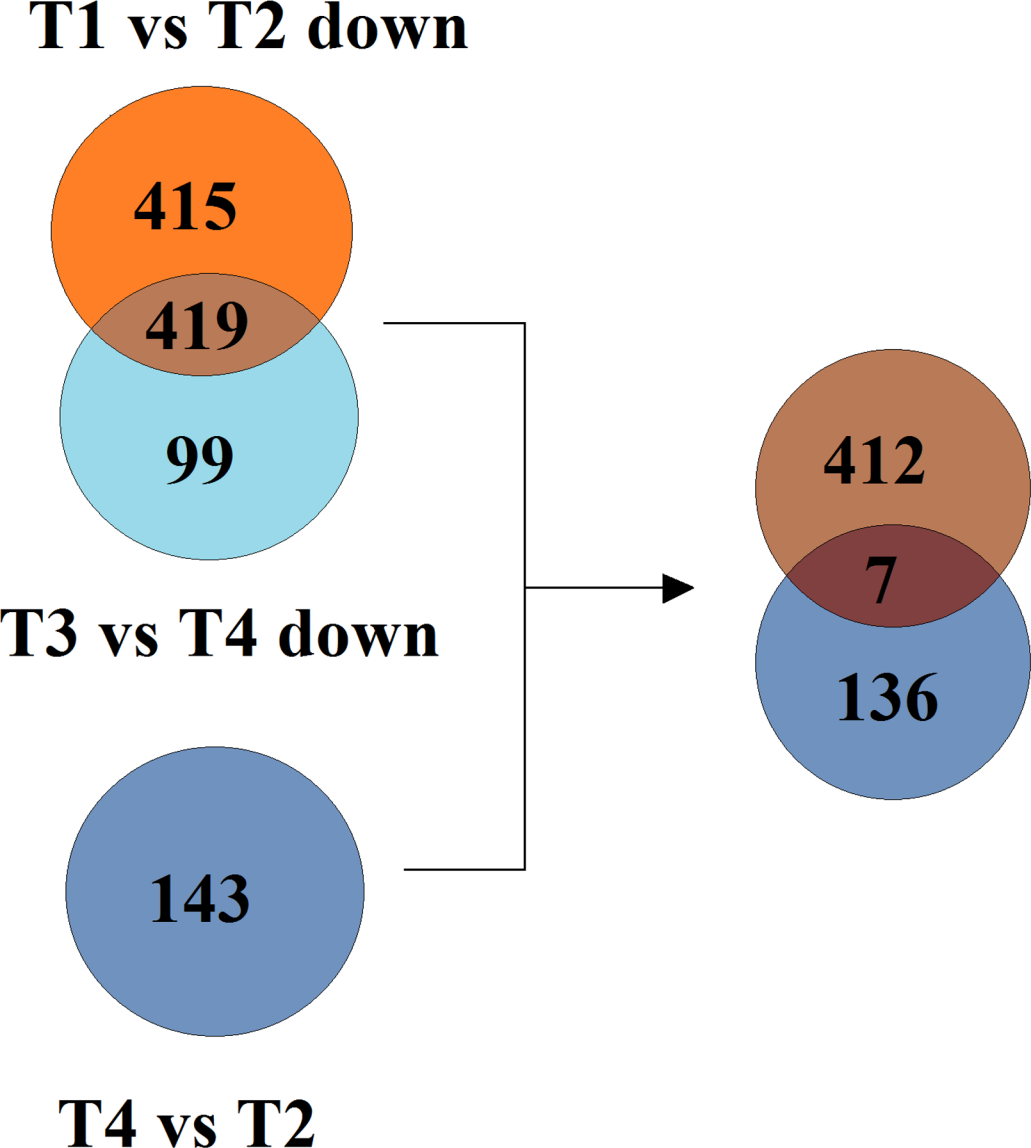
Screening of the DEGs that specifically expressed in young spikes. T1, stem tips of D; T2, spikes of D; T3, stem tips of T; T4, spikes of T.

To screen out spike specifically expressed genes in the 143 DEGs (S3 Table), they were compared with the 419 genes significantly highly expressed in both spikes of T and D (Fig 9). Seven specifically expressed DEGs in spikes were obtained (Table 5). The homologic gene search in NCBI indicated that the unigene T2-63525 was the gene of dbj|AK334518.1| from Chinese Spring, which was a gene specifically expressed in inflorescence (http://www.ncbi.nlm.nih.gov/UniGene/ESTProfileViewer.cgi?uglist=Ta.67718). The dynein light chain *LC6* like gene family was mainly expressed in wheat spike and stem (http://www.ncbi.nlm.nih.gov/UniGene/ESTProfileViewer.cgi?uglist=Ta.21814). Noticeably, five of the seven genes belonged to biological process of cell cycle control, cell division and chromosome partitioning. Three of the five genes were wheat dynein light chain *LC6* (*TaLC6*). Furthermore, the expressions of all the three *TaLC6* genes were significantly lower. It should be an important gene family that played a fundamental role in differentiating spikes.

**Table 5:** Seven DEGs highly expressed in young spikes.

Functional class of genes	Unigene Id	Transcripts (T4/T2)*	Putative or homologic protein**
Cell cycle control, Cell division	T1-47968	112/48	Dynein light chain LC6 *(B*. *distachyon*)
	T2-40114	164/67	Dynein light chain LC6
	T4-51695	137/57	Dynein light chain LC6
Chromosome partitioning	T2-63525	45/161	Predicted protein F775_08983 (*A*. *tauschii*)
	T4-15960	46/103	TRIUR3_08346 (*T*. *urartu*)
Translation	T4-34116	128/21	Ubiquitin-60S ribosomal protein L40-2 (T. urartu)
	T4-61565	130/264	Translation initiation factor IF-2 (*O*. *sativa*)

* T2, spikes of D; T4, spikes of T.

** *B*. *distachyon*: *Brachypodium distachyon*; *A*. *tauschii*: *Aegilops tauschii*; *T*. *urartu*: *Triticum urartu*; *O*. *sativa*: *Oryza sativa*.

The functions of the DEGs were annotated referred to seven databases (Table 6). Twenty-six out of the 143 DEGs were involved in wheat differentiation and development according to the functional annotation referred to GO (Table 4; S3 Table).

**Table 6:** Functional classification of DEGs in young spikes.

Functional class of DEGs	Total number of genes	Up-regulated genes	Down-regulated genes
**Cellular component**	10	6	4
**Energy production and conversion**	7	0	7
**Replication, recombination and repair**	3	2	1
**Transcription**	13	4	9
**Translation, ribosomal structure and biogenesis**	11	3	8
**Posttranslational modification, protein turnover, chaperones**	4	3	1
**Amino acid transport and metabolism**	9	5	4
**Signal transduction mechanisms**	5	5	0
**Response to out stimulus**	14	2	12
**Cell cycle control, cell division, chromosome partitioning**	6	0	6
**Photosynthesis**	10	0	10
**Carbohydrate transport and metabolism**	10	1	9
**Plastid**	4	3	1
**Lipid transport and metabolism**	4	3	1
**Inorganic ion transport and metabolism**	1	1	0
**General function**	6	4	2
**Unkown**	26	7	19

**Fig 10:**
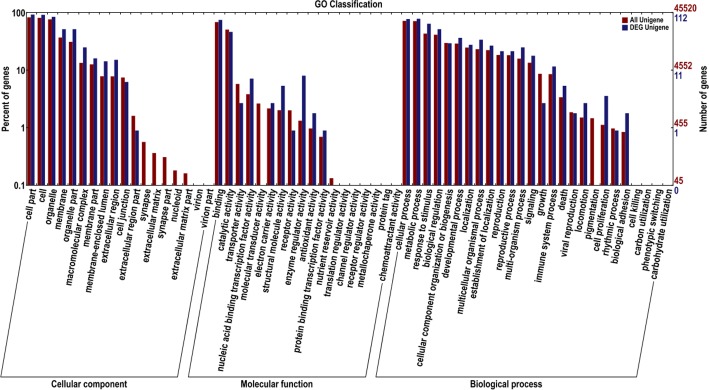
Functional classification of DEGs in differentiating spikes.

Functional classification of the DEGs in differentiating spikes of T and D were carried out referred to databases COG and GO (Fig 10). The results indicated that, (1) cellular components of extracellular region part, extracellular matrix and extracellular matrix part were significantly changed; (2) molecular functions of transporter, nucleic acid binding transcription factor, molecular transducer, structural molecule, receptor, enzyme regulator, antioxidant and nutrient reservoir activity were significantly changed; (3) biological processes of response to stimulus, biological regulation, developmental process, multicellular organismal, multi-organism, growth, immune system, death, locomotion, pigmentation, cell proliferation, and biological adhesion were significantly changed (Fig 10).

Regarding young spikes of D as control, KEEG pathway enrichment analysis indicated that 23 pathways were up regulated (many DEGs in the pathway were up regulated), 7 pathways were down regulated, 3 pathways had up and down regulated DEGs in differentiating spikes of T (Tables 6 and 7). Based on the computer analyses of the DEGs referred to databases, manual analysis was also carried out (Table 4; S3 Table). These data demonstrated that mutant significantly repressed some major metabolism pathways, that especially were photosynthesis—antenna proteins, photosynthesis, carbon fixation in photosynthetic organisms, glycolysis / gluconeogenesis, galactose metabolism, fructose and mannose metabolism, these were close associated with production of ‘energy’ and ‘construction materials’ for plant differentiation and growth.

**Table 7:** KEEG pathway enrichment analysis of the DEGs.

Pathway	DEGs with pathway annotation (32)	All genes with pathway annotation (5389)	*P* value	Corrected *P* value	Pathway ID*
Photosynthesis—antenna proteins	4 (12.5%)	31 (0.58%)	2.88E-05	9.51E-04	ko00196
Glycolysis / Gluconeogenesis	7 (21.88%)	205 (3.8%)	1.56E-04	5.13E-03	ko00010
Carbon fixation in photosynthetic organisms	4 (12.5%)	101 (1.87%)	2.79E-03	9.22E-02	ko00710
Photosynthesis	3 (9.38%)	79 (1.47%)	1.11E-02	3.66E-01	ko00195
Alanine, aspartate and glutamate metabolism	3 (9.38%)	80 (1.48%)	1.15E-02	3.78E-01	ko00250
Pentose phosphate pathway	3 (9.38%)	80 (1.48%)	1.15E-02	3.78E-01	ko00030
Galactose metabolism	3 (9.38%)	87 (1.61%)	1.44E-02	4.75E-01	ko00052
Fructose and mannose metabolism	3 (9.38%)	92 (1.71%)	1.67E-02	5.51E-01	ko00051
Nitrogen metabolism	2 (6.25%)	82 (1.52%)	8.46E-02	1	ko00910
Taurine and hypotaurine metabolism	1 (3.13%)	23 (0.43%)	1.28E-01	1	ko00430
Limonene and pinene degradation	1 (3.13%)	24 (0.45%)	1.33E-01	1	ko00903
Stilbenoid, diarylheptanoid and gingerol biosynthesis	1 (3.13%)	24 (0.45%)	1.33E-01	1	ko00945
Pyruvate metabolism	2 (6.25%)	120 (2.23%)	1.59E-01	1	ko00620
Ascorbate and aldarate metabolism	1 (3.13%)	1 (3.13%)	2.27E-01	1	ko00053
Biosynthesis of unsaturated fatty acids	1 (3.13%)	44 (0.82%)	2.31E-01	1	ko01040
Fatty acid biosynthesis	1 (3.13%)	45 (0.84%)	2.36E-01	1	ko00061
Tyrosine metabolism	1 (3.13%)	58 (1.08%)	2.93E-01	1	ko00350
Propanoate metabolism	1 (3.13%)	58 (1.08%)	2.93E-01	1	ko00640
Tryptophan metabolism	1 (3.13%)	60 (1.11%)	3.02E-01	1	ko00380
Protein processing in endoplasmic reticulum	2 (6.25%)	218 (4.05%)	3.74E-01	1	ko04141
Citrate cycle (TCA cycle)	1 (3.13%)	79 (1.47%)	3.77E-01	1	ko00020
Fatty acid metabolism	1 (3.13%)	79 (1.47%)	3.77E-01	1	ko00071
Inositol phosphate metabolism	1 (3.13%)	81 (1.5%)	3.85E-01	1	ko00562
Plant hormone signal transduction	2 (6.25%)	245 (4.55%)	4.31E-01	1	ko04075
Peroxisome	1 (3.13%)	97 (1.8%)	4.42E-01	1	ko04146
Arginine and proline metabolism	1 (3.13%)	97 (1.8%)	4.42E-01	1	ko00330
Spliceosome	2 (6.25%)	287 (5.33%)	5.15E-01	1	ko03040
Amino sugar and nucleotide sugar metabolism	1 (3.13%)	123 (2.28%)	5.23E-01	1	ko00520
Cysteine and methionine metabolism	1 (3.13%)	124 (2.3%)	5.26E-01	1	ko00270
Plant-pathogen interaction	1 (3.13%)	145 (2.69%)	5.83E-01	1	ko04626
Ribosome	2 (6.25%)	332 (6.16%)	5.95E-01	1	ko03010
Ubiquitin mediated proteolysis	1 (3.13%)	165 (3.06%)	6.31E-01	1	ko04120
Endocytosis	1 (3.13%)	190 (3.53%)	6.84E-01	1	ko04144

* Number of the metabolism pathway in Kanehisa Laboratories.

### Expression profiles of twelve genes in differentiating spikes

To validate the reliability of the transcriptome sequencing results, and further explore the expression profiles of the important DEGs (Table 4), twelve DEGs considered as important genes involved in wheat spike differentiation were selected first to do expression profiling in spikes of D, M, T and ‘Zhoumai 18’ (Table 4, marked with ‘*’). The change directions of the gene expression levels at the early stage of floral organ development were completely in agreement with the results of transcriptome sequencing, which confirmed the sequencing results truly reflected the facts. Some gene expression fluctuated significantly during wheat spike developmental phases, which showed the spatio-temporal character of their function (Fig 7A–7L. A: unigene T1-38135; B: T1-47968 (*TaLC6*); C: T1-49256; D: T1-56016; E: T1-71202; F: T2-14438; G: T2-41011; H: T2-47003; I: T2-47331; J: T4-20296; K: T4-52638; L: T4-58706. Functional annotation of the unigenes (their homologs or similar genes) see Table 4. The *actin* gene was used as internal control. The number 1–16 indicate the sampling dates, and the details see S1 Table.).

Mutant significantly suppressed the expression of a putative alpha-L-fucosidase 1-like gene (T4-52638, K), however it was highly expressed in T, M and ‘Zhoumai 18’, reached to several ten thouthand (control gene *grdph*) or several hundred thousand times (control gene *actin*) compared with the internal control genes, that suggested its transcript was abundance and it played a fundamental role during spike development. Mutation suppressed the expression of a replication factor A protein 1-like gene (T4-58706, L); suppressed an unkown gene (T2-14438, F). Mutation significantly activated the expressions of a homeobox-leucine zipper protein HOX24-like gene (T1-56016, D); an amidase 1-like gene (T2-41011, G); a BTB/POZ and TAZ domain-containing protein 2-like gene (T2-47003, H); and a serine / arginine repetitive matrix protein 1-like gene (T2-47331, I). The seven genes almost exclusively expressed in D or absolutely did not express in D. The contrary expression profiles compared with that of T (as well as M and ‘Zhoumai 18’) suggested their very important roles in wheat development.

The expression of a heat shock protein 81-1-like gene was increasing with spike development, its expression level was relatively lower in D (T1-38135, A). *TaLC6* was suppressed at early stage in D, but highly expressed at later stage (T1-47968, B). A gene broadly similar to an uncharacterized 35.5 kDa protein in transposon Tn4556 was an unstable expressed gene (T1-49256, C). A probable alpha, alpha-trehalose-phosphate synthase gene was lower expressed at early stage and highly expressed at later stage in D (T1-71202, E). An UDP-glucose 4-epimerase 3-like gene was highly expressed at early middle stage in T and later in D (T4-20296, J). The expression profiles of this five genes were different between D and other three genotypes, but not sharp as that of the above seven genes. Their expressions were also significantly effected by mutation, so they might also play important roles during spike development.

## Discussion

### Super-bulks at appropriate developmental stages guaranteed a reliable result

Compared with cDNA microarray [30], next generation sequencing (NGS) technologies are high throughput and more direct approaches for fine-mapping and gene isolation in many species [31], but sequencing technology can not eliminate biological variability [32]. An elaborately designed RNA-sequencing experiment can eliminate biological variability and produce reproducible results. In this paper, mRNA sequencing combined with super bulked segregant analysis (S-BSA) successfully profiled the whole transcriptomes of differentiating spikes on D and T plants of a wheat mutant *dms*.

The genes specifying the original meristem of stamens and pistils should express at stage of pistil / stamen primordium, thus we pooled 20 individual spikes in this phase for each super-bulked sample, which was more than that in most researches with only one or several individuals [32]. The transcriptomes of spikes and their stem tips of both T and D were parallel experiments in fact (Fig 6). Thus the approach not only guaranteed the key genes for floral organ differentiation were in the pools, but also mostly eliminated the confounding of the background genes and biological variability. A total 143 significant DEGs were screened out between D and T genotypes, which was smaller than that of the similar studies [30]. However, the expression profiling of the twelve genes completely confirmed the results of the RNA sequencing without exception (Fig 7A–7L. A: unigene T1-38135; B: T1-47968 (*TaLC6*); C: T1-49256; D: T1-56016; E: T1-71202; F: T2-14438; G: T2-41011; H: T2-47003; I: T2-47331; J: T4-20296; K: T4-52638; L: T4-58706. Functional annotation of the unigenes (their homologs or similar genes) see Table 4. The *actin* gene was used as internal control. The number 1–16 indicate the sampling dates, and the details see S1 Table). Furthermore, most expression profiles of the homeotic genes in D and T were highly consistent (Fig 5), these demonstrated the sequencing was successful. The global expression profiles in this study truly reflected the characteristics of the mutant, and provided a set data of transcriptomes for further studies.

### Elements specify wheat floral organ and influence spike development

We identified 419 genes highly expressed in wheat differentiating spikes, which highlight the elements specifying floral organ and influencing spike development.

#### Homeotic genes specify wheat floral organ

We have identified eight MADS-box genes in this study, six were significantly highly or exclusively expressed in differentiating spikes, they were homologs of *OsMADS6* [33], *OsMADS15* [33], *OsMADS18* [33], *OsMADS32* [34, 35] *an*d *OsMADS34* [34, 35] (Table 3, Fig 5). Transition from the vegetative to the reproductive growth phase in winter wheat needs vernalization, that is determined by allelic differences in the MADS-box transcription factor *VERNALIZATION1* [32], which is closely related to the three paralogous *Arabidopsis* floral meristem identity genes *APETALA1* (*AP1*), *CAULIFLOWER* (*CAL*) and *FRUITFULL* (*FUL*). *VRN1* is also homologous to *FUL2* (*= OsMADS15*) and *FUL3* (= *OsMADS18*), *VRN3* is homolog of *FLOWERING LOCUS T* (*FT*) in *A*. *thaniala* [36]. In rice, a MADS-box gene *PANICLE PHYTOMER2* (*PAP2* = *OsMADS34*) combined with the *AP1/FUL2/FUL3* homologs were needed for the transition of the shoot apical meristem to the reproductive stage [37]. Coexpression meta-network of *OsMADS34* in rice and knock-out phenotypes characterized in surrounding network for *OsMADS34* indicate its key role in spike development. The *OsMADS6* is *AGAMOUS-LIKE6* (*AGL6*) gene, which regulates floral organ identity and floral meristem determinacy [38]. The *WFUL2* (wheat *FUL*-like genes) of wheat has class A functions in specifying the identities of floral meristems and outer floral organs (lemma and palea) through collaboration with class B and class E genes [39]. The stem tips used in this study were very short fragments connected with the differntiating spikes (Fig 2), and the homologs of *OsMADS34* (*TaSEP-5*), *OsMADS6* (*TaAGL6*) and *OsMADS15* (*TaAP1-3*) almost exclusively expressed in differentiating spikes (Fig 5). The spike specific expression patterns demonstrated the six MADS-box genes played determining roles in wheat floral organ differentiation.

Two homologs of *O*. *sativa AP2*-like ethylene-responsive transcription factor gene (= *AINTEGUMENTA*, *ANT*), one homolog of *O*. *sativa* transcription factor *FLORICAULA/LEAFY* (*FL*), and one homolog of *O*. *sativa* protein *DROOPING LEAF* (*DL*) were identified in this study (Table 3), they all were significantly highly expressed in differentiating spikes (Fig 5). The *AP2/ETHYLENE RESPONSE FACTOR* (*ERF*) transcription factor genes are typically expressed in the organ primordia that they help to specify floral organ [40]. In *Arabidopsis*, integuments depend on the expression of the *AP2 ⁄EREBP* transcription factor *ANT*. The *FL* genes encode plant-specific transcription factors and play major roles in the reproductive transition as well as floral organ development [41–43]. In grasses the homeotic function for carpel differentiation is attributed to a YABBY domain gene named *DROOPING LEAF* (*DL*) [44–47]. The expression patterns suggested that the homologs of *ANT*, *FL* and *DL* identified in this study were also key factors for wheat floral organ differentiation (Fig 5).

Floral organ development studies indicate that most genes involved in the differentiation of floral meristem and organ identity and in the transition to flowering in some model plants such as *Arabidopsis*, *Antirrhinum*, and *Petunia* belong to conserved and widespread gene families encoding transcription factors, such as the MADS-box genes, *FLO*-like, and *AP2*-like genes [48]. Currently, the ABCDE model for flower development proposes that floral organ identity is defined by five classes of homeotic genes, named A, B, C, D and E, class A genes specify the identity of sepals, class A and B genes specify petal identity, class B and C genes determine stamen identity, and class C genes determine carpel identity, class D genes are crucial for ovule development, class E genes are required for the differentiation of all kinds of floral organs [49, 50]. Most homeotic genes are conserved in grasses [51].

The studies on floral organ differentiation in wheat lagged behind that of many plant species such as *Arabidopsis* and *O*. *sativa*. However, some researches have been carried out to interpret the floral organ development in wheat employing materials with spike abnormality of pistillody [29]. The pistillody in cytoplasmic substitution (alloplasmic) lines of bread wheat has been suggested to be induced by mitochondrial retrograde signaling. The fertility-restoring dominant gene *Rfd1* inhibits pistillody and female sterility. The B MADS-box genes, wheat *APETALA3* (*WAP3*) [52], two wheat *PISTILLATA* (*PI*) orthologs, wheat *PISTILLATA-1* (*WPI-1*) and *WPI-2* [53], *WANT-1*, a wheat *ANT* homolog [54], a wheat *SEEDSTICK* (*WSTK*) -like class D MADS-box gene [55], a wheat pistillody-related protein kinase 1 (*WPPK1*) [56], a wheat ortholog of *DROOPING LEAF* (*TaDL*) [51], a wheat *Calmodulin-Binding Protein 1* (*WCBP1*) [19], and a mitochondrial gene *orf260* [57] are associated with the pistillody. These data suggested that most homeotic genes involved in floral organ development of wheat, and the pistillody was a consequence of a complex GRNs’ alteration.

Similarly, using a multi-pistil and male sterility mutant *dms*, we demonstrated wheat homologs of MADS-box *MFO1* (E class), *FUL2* (A class), *FUL3* (A class), *PAP2* (E class) etc, *AP2* (A class), *FL* and *DL* (CRC) genes played determining roles at early stages of floral organ differentiation in wheat. This was confirmed in some extent by temporal expression profiles of many homeotic genes during spike development of D, M and T plants (unpublished data). Combined with other researcher’s results [29, 39], the representative genes of all A, B, C, D, E classes have been identified in wheat. Although diversity may exist, it is clear that wheat floral organ differentiation also be determined following ABCDE model.

#### Other gene expression regulators play important roles in wheat floral organ development

In addition to transcription factors of typical homeotic genes described above, we identified some other DNA replication, transcription and translation factor genes significantly highly expressed in differentiating spikes (Table 3). According to GO annotation, they are involved in plant floral organ development. For example, E3 ubiquitin-protein ligase, mostly expressed in inflorescence. *AtPUB4*, a U-box/ARM repeat-containing E3 ubiquitin ligase, is a novel player in male fertility in *Arabidopsis*. Loss of *AtPUB4* function causes hypertrophic growth of the tapetum layer. The *Atpub4* mutation also leads to incomplete degeneration of the tapetal cells and strikingly abnormal exine structures of pollen grains [58]. E3 ubiquitin ligase participates in CpG methylation-dependent transcriptional regulation and epigenetic transcriptional silencing [59]. BTB/POZ (Bric-a-brac, Tramtrack, and Broad Complex/Pox virus and Zinc finger) domain-containing protein NPY1 (= ENP, MAB4) may act as a substrate-specific adapter of an E3 ubiquitin-protein ligase complex (CUL3-RBX1-BTB) which mediates the ubiquitination and subsequent proteasomal degradation of target proteins. It expresses in the inflorescence meristem and at various stages of floral development. Disruption of *NPY1* does not cause obvious defects in organogenesis, but *npy1 npy3 npy5* triple mutants failed to make flower primordia, *NPY* genes define one key step in a pathway that controls YUCCA1 (YUC)-mediated organogenesis in *Arabidopsis* [60]. WD-40 repeat-containing protein MS14 (= ACG1, FVE; = Altered cold-responsive gene 1 protein; *cag*) is a core histone-binding subunit that may target chromatin assembly factors, chromatin remodeling factors and histone deacetylases to their histone substrates in a manner that is regulated by nucleosomal DNA. It’s a component of the flowering autonomous pathway which positively regulates flowering by promoting transcriptional repression of the flowering repressor *FLOWERING LOCUS C (FLC)* [61]. The homologs of DNA (cytosine-5)-methyltransferase and E3-ubiquitin-protein ligase ORTHRUS 2 indicated that methylation played a role in floral organ development. The BTB/POZ domain-containing protein 2-like gene was suppressed at early stage and highly expressed at later stage in D (Fig 7H. A: unigene T1-38135; B: T1-47968 (*TaLC6*); C: T1-49256; D: T1-56016; E: T1-71202; F: T2-14438; G: T2-41011; H: T2-47003; I: T2-47331; J: T4-20296; K: T4-52638; L: T4-58706. Functional annotation of the unigenes (their homologs or similar genes) see Table 4. The *actin* gene was used as internal control. The number 1–16 indicate the sampling dates, and the details see S1 Table). The homologs of these genes (E3 ubiquitin ligase, BTB domain-containing *NPY1*, *FVE* etc) identified in this study suggested that except homeotic genes, there were many other ‘gene expression’ regulators and protein interaction genes involved in wheat floral organ development.

#### Carbohydrate metabolism plays important roles in wheat floral organ development

Carbohydrate metabolism related genes consisted of another large group genes in the DEGs involved in wheat spike differentiation (Table 3). Most of them were involved in cell wall biogenesis and degradation, including various endoglucanase, polygalacturonase inhibitor and glucan endo-1, 3-β-glucosidase. The homologs of endoglucanase (OsGLU8, OsCel9E, OsGLU13) were endo-1, 4-β-glucanase (EC 3.1.2.4), endohydrolysis of (1–4)-β-D-glucosidic linkages in cellulose, lichenin and cereal β-D-glucans. A glycoside hydrolase family (GHF) 9 gene of rice, *OsCe19A*, corresponding to the auxin-induced 51 kDa endo-1, 4-β-glucanase (EGase). This enzyme reveals a broad substrate specificity with respect to sugar backbones (glucose and xylose) in β-1, 4-glycans of type II cell wall [62]. Polygalacturonase inhibitor (Polygalacturonase-inhibiting protein, PGIG) is an inhibitor of fungal polygalacturonase. Glucan endo-1, 3-β-glucosidase (EC 3.2.1.39) hydrolysis of (1–3)-β-D-glucosidic linkages in (1–3)-β-D-glucans, lysis of cellular walls containing β-1, 3-glucans, and is implicated in the defense against fungal pathogens, starch and sucrose metabolism and development [63]. This indicated the cell wall biogenesis / degradation was more active and important for differentiating spikes.

#### Phytohormone and other signaling pathways influence wheat spike differentiation

Phytohormone is a kind of very important regulator for plant floral organ development. For example, *GIBBERELLIC ACID REQUIRING 1* (*GA1*) was associated with the length of petals, stamens, and to a lesser extent style-stigma length [64]. *AP2* is a typical homeotic gene. We identified two *AP2*-like genes, one auxin response factor 14-like gene highly expressed in differentiating spikes. The relationship between stem development and GA metabolism of the wheat mutant *dms* was studied by measuring GA concentrations in the upper internodes of *dms* using High Performance Liquid Chromatography (HPLC), and investigating the effects of exogenous GA_3_ on plant height in field and coleoptile length in lab (the data will be published elsewhere). It confirmed the GA signal pathway in D plants was different from that of T plants. These data suggested that phytohormone signaling was important when spike development in wheat also.

We identified two LRR receptor-like serine / threonine-protein kinase (RLK) genes highly expressed in differentiating spikes (Table 3). LRR-RLK is a large group important signalling molecules involved in various biological processes. In *Arabidopsis*, LRR-RLK ERECTA (= ER, QRP1, QRS1, TE1) strongly expressed in organ primordia and immature organs but weakly in mature organs. It was observed in shoot apical meristems (SAM) at low levels during the vegetative growth with an increase at the transition to the reproductive growth phase. At the reproductive stage, localized in the young developing flowers, expressed in inflorescence meristem and is up-regulated during flower initiation and formation of flower organs. Receptor kinase that, together with ERL1 and ERL2 (two functional paralogs of ERECTA), regulates aerial architecture, including inflorescence. Loss of the entire ERECTA family genes led to striking dwarfism, reduced lateral organ size and abnormal flower development, including defects in petal polar expansion, carpel elongation, anther and ovule differentiation. These defects are due to severely reduced cell proliferation [65]. The LRR-RLK genes in ERECTA family, as well as the zinc finger protein genes identified in this study may be involved in the GRNs of wheat floral organ differentiation.

### Molecular basis for abnormal spike differentiation of mutant *dms*

In this study, we found mutation significantly affected the expressions of homeotic genes, many transcription factors, signaling related genes, phytohormone response genes, cell division related genes, carbohydrate transport and metabolism, protein synthesis and photosynthesis related genes (Table 4; S3 Table). These were the molecular basis for abnormal spike differentiation of the mutant *dms*.

#### Two class A homeotic genes were suppressed in D

Homeotic genes play important roles in wheat transition from vegetative to floral organ growth [66]. Noticeably, the *TaAP1-2* was highly expressed in spikes of T, but no detected in D. The *TaAP2* was significantly highly expressed in spikes of T than in D (Fig 5). Both of *AP1* and *AP2* were key floral organ identity genes of class A [36, 67]. Murai [29] postulated that the mutual suppression between class A and C genes existed in wheat. Class A genes determine palea and lodicule development, class C genes determine the steman and pistils. The expressions of class A genes *TaAP1-2* and *TaAP2* were inhibited that possibly activated the class C genes, and resulted in forming additional pistils.

#### Other transcription factors

Most biological processes in a eukaryotic cell or organism are finely controlled at the transcriptional level by transcription factors [68]. Genome-wide studies have revealed that many of the genes that are regulated by the floral organ identity factors encode transcriptional regulators. In *Arabidopsis* the percentage of transcription factor-coding genes identified in microarray experiments on floral tissues is 5.5% ~ 26% of DEGs [40]. In this study, transcription factor-coding genes consisted of a big group of DEGs in mutant *dms* (23 unigenes, 16.08% of DEGs; S3 Table). The transcription factors are complex. The following were some of them.

BTB/POZ domain scaffold proteins are present in many organisms. BTB-containing proteins are numerous and control cellular processes that range from actin dynamics to cell-cycle regulation. In spite of their high sequence divergence and apparently unrelated functions, many BTB-containing proteins have at least one shared role: the recruitment of degradation targets to E3 ubiquitin ligase complexes [69]. In *Arabidopsis* they form a family of eighty proteins that have been implied in a variety of signaling pathways. The BTB and TAZ (transcriptional adapter zinc finger) domain protein 1 (BT1) is interactor and regulator of the PINOID (PID) kinase. BT1 has a typical domain structure that is only observed in land plants. The *Arabidopsis BT* family, which comprises five members, named BT1 to BT5, of which at least four are encoded by auxin responsive genes. There is considerable functional redundancy among the *BT* members, and that BT function is crucial for early steps in both male and female gametophyte development. BT proteins, as regulators of PID activity, are likely part of a feed-back loop that affects auxin distribution [70]. The expresstion of a BTB/POZ and TAZ domain-containing protein 2-like gene (T2-47003) in spikes of D was significantly suppressed at early stage, but highly expressed at later stage (Fig 7H. A: unigene T1-38135; B: T1-47968 (*TaLC6*); C: T1-49256; D: T1-56016; E: T1-71202; F: T2-14438; G: T2-41011; H: T2-47003; I: T2-47331; J: T4-20296; K: T4-52638; L: T4-58706. Functional annotation of the unigenes (their homologs or similar genes) see Table 4. The *actin* gene was used as internal control. The number 1–16 indicate the sampling dates, and the details see S1 Table). It may be interacted with auxin signal pathway as most *BT* genes in *Arabidopsis*.

The *ASYMMETRIC LEAVES2* (*AS2*) gene is required for the generation of the flat and symmetrical shape of the leaf lamina in *Arabidopsis*. *AS2* encodes a plant-specific protein with an AS2/LATERAL ORGAN BOUNDARIES (AS2/LOB) domain that includes a cysteine repeat, a conserved single glycine residue and a leucine-zipper-like sequence in its amino-terminal half. Some members of the AS2/LOB family might have redundant function(s). The AS2/LOB domain of AS2 cannot be functionally replaced by those of other members of the family, and that only a few dissimilarities among respective amino acid residues of the AS2/LOB domain of AS2 and those of other members are important for the specific functions of AS2 [71]. The expression of a LOB domain-containing protein 37-like gene was significantly suppressed in spikes of D. The functional annotation implied it could regulate transcription.

Four LOC284861-like genes (T2-16245, T1-64318, T4-61132 and T2-31732) were significantly suppressed in D, unigene T2-16245 almost only expressed in T, suggested a very important role in wheat floral organ differentiation. Uncharacterized protein LOC284861 is a new protein of *Homo sapiens*. Blastn indicated that the unigene T2-16245 was moderately similar to a sequence (emb/FN564432.1) in wheat 3B-specific BAC library by 70% identity over 354 bp region. Such, unigene T2-16245 was a novel gene in wheat. The functional annotation implied it could regulate transcription, and its function needs to be explored in future.

#### Phytohormone signalling

Abscisic acid (ABA), auxin, ethylene, and gibberellin signaling related genes were affected in mutant, the later two might be more important for floral organ development, because *AP2* [40, 48, 72], and *GA1* [60] were certainly function in plant floral organ development. The expressions of two auxin-repressed 12.5 kDa protein genes were up-regulated in D, indicated that the concentration of auxin in the spikes of D might be lower, and auxin pathway was repressed. The dioxygenase for auxin oxidation *(DAO)* gene, encoding a putative 2-oxoglutarate-dependent-Fe (II) dioxygenase, is essential for anther dehiscence, pollen fertility, and seed initiation in rice [73]. This indicated the important role of auxin signaling in floral organ development. Repressed auxin signal pathway would decrease cell division and proliferation, thereby affected growth. An ABA inducible protein PHV A (cold acclimation protein WCOR615) was suppressed, one gibberellin receptor *GID1*, two gibberellin stimulated transcript (*GAST1*; = Gibberellin Stimulated-Like proteins 1, *GSL1*, = *snakin-1*) genes were up-regulated in D. In tomato (*Lycopersicon esculentum* Mill.), gibberellic acid (GA3) increases the abundance of GAST1 RNA while treatment with ABA blocks this effect [74]. In *Arabidopsis*, GASA5 suppresses GA responses, GA induces or suppresses the transcription of different *GAST1-*like genes and the encoded proteins regulate the redox status of specific components to promote or suppress specific GA responses [75]. Potato (*Solanum tuberosum* L.) *GSL1* and *GSL2* genes are very highly conserved. Potato transformation with antisense knock-down expression cassettes failed to recover viable plants (lethality; similar to D genotype in this study in some extent). GSL protein family plays a role in several aspects of plant development [76]. Gibberellin pathway plays an important role in wheat transition from vegetative to floral growth [66]. The relationship among gibberellin, *VRN1*, *FT*, *SUPPRESSOR OF OVEREXPRESSION OF CONSTANS1-1* and *FL* in determining wheat flowering has been studied. GA and *FL* play critical roles during early stages of wheat flower development [77]. These cases indicate that the balance of hormone signalings is pivotal for floral organ differentiation. In this study, the expressions of *GID1* and *GAST1*-like genes were significantly activated, indicated that the gibberellin signaling pathway was significantly altered. Generally, the signal pathways of ABA, ethylene and auxin were inhibited in D, but gibberellin was activated, thereby resulted in hormonal imbalance. We have primarily confirmed that GA signaling in D was different from T and M plants (the data will be published elsewhere). The phytohormone regulation in D was very complex, but hormonal imbalance should be one aspect.

#### Carbohydrate transport and metabolism were significantly suppressed in D

Combined with photosynthesis, energy production and conversion, this was the biggest group (27 unigene, 18.88% of DEGs, S3 Table) of DEGs between T and D. Carbohydrate not only provides the energy for various biological processes, but also provides materials for cell wall construction. Carbohydrate transport and metabolism were significantly suppressed in D. For example, the expressions of putative alpha-L-fucosidase 1 and mitochondrial outer membrane protein porin 1-like genes were almost inhibited completely (Fig 7K. A: unigene T1-38135; B: T1-47968 (*TaLC6*); C: T1-49256; D: T1-56016; E: T1-71202; F: T2-14438; G: T2-41011; H: T2-47003; I: T2-47331; J: T4-20296; K: T4-52638; L: T4-58706. Functional annotation of the unigenes (their homologs or similar genes) see Table 4. The *actin* gene was used as internal control. The number 1–16 indicate the sampling dates, and the details see S1 Table). Alpha-L-fucosidase 1 is an element of carbohydrate metabolism. The transcripts of alpha-L-fucosidase 1-like gene were abundance. Its expression was sharply suppressed in D (Fig 7K. A: unigene T1-38135; B: T1-47968 (*TaLC6*); C: T1-49256; D: T1-56016; E: T1-71202; F: T2-14438; G: T2-41011; H: T2-47003; I: T2-47331; J: T4-20296; K: T4-52638; L: T4-58706. Functional annotation of the unigenes (their homologs or similar genes) see Table 4. The *actin* gene was used as internal control. The number 1–16 indicate the sampling dates, and the details see S1 Table), thus we considered it as an important gene influencing wheat floral organ differentiation. The cell wall biogenesis / degradation were more active and important for differentiating spikes [62, 63]. The male sterility induced by a gametocide SQ-1 might be associated with imbalance of energy metabolism [78]. The wheat line 3558 has two type tillers of inhibited and normal. Development was blocked or delayed at the elongation stage in the inhibited tillers of 3558. Weakened energy metabolism might be one reason that the inhibited tillers could not joint and develop into spikes [79]. The plants of D were very short (<30 cm) and completely sterile. The suppressed carbohydrate transport and metabolism, photosynthesis, energy production and conversion, should have played a basic role for morphological abnormalities of D.

#### Significant DEGs need investigation in detail

Some genes significantly differently expressed in spikes of D and T had been picked out through mRNA sequencing and QRT-PCR analysis. The expressions of an unknown gene in 1Ds prolamin gene locus (retrotransposon protein), mitochondrial outer membrane protein porin 1, replication factor A protein 1, uncharacterized protein LOC284861, transcription-repair-coupling factor, lysine-rich arabinogalactan protein 19, ABA-inducible protein PHV A1, putative alpha-L-fucosidase 1, uncharacterized protein DKFZp434B061, splicing factor 3A subunit 1-like, setaria italica titin-like, hypothetical protein F775_20975, G-protein coupled receptor 114-like and others without any similar known gene or sequences in GenBank were almost completely inhibited in D. The expressions of a amidase 1, bowman-birk type wound-induced proteinase inhibitor WIP1, homeobox-leucine zipper protein HOX24, serine/arginine repetitive matrix protein 2, metallothionein-like protein 1, LEA protein 12, isoflavone 2’-hydroxylase, probable WRKY transcription factor 70-like gene, and others without any similar known gene or sequences in GenBank were significantly up-regulated in D (Table 4; Fig 7A–7L. A: unigene T1-38135; B: T1-47968 (*TaLC6*); C: T1-49256; D: T1-56016; E: T1-71202; F: T2-14438; G: T2-41011; H: T2-47003; I: T2-47331; J: T4-20296; K: T4-52638; L: T4-58706. Functional annotation of the unigenes (their homologs or similar genes) see Table 4. The *actin* gene was used as internal control. The number 1–16 indicate the sampling dates, and the details see S1 Table). Although some of them were absolutely novel genes in wheat without any putative functions, the entirely opposite expression patterns between T and D implied the important roles they played in wheat floral organ development, and they were the major causal factors for spike abnormality of D (Fig 7A–7L. A: unigene T1-38135; B: T1-47968 (*TaLC6*); C: T1-49256; D: T1-56016; E: T1-71202; F: T2-14438; G: T2-41011; H: T2-47003; I: T2-47331; J: T4-20296; K: T4-52638; L: T4-58706. Functional annotation of the unigenes (their homologs or similar genes) see Table 4. The *actin* gene was used as internal control. The number 1–16 indicate the sampling dates, and the details see S1 Table). For example, the unknown gene T2-14438, similar to a hypothetical protein F775_20975 of *Aegilops tauschii*, whose expression was significantly suppressed in spikes of D (Fig 7F. A: unigene T1-38135; B: T1-47968 (*TaLC6*); C: T1-49256; D: T1-56016; E: T1-71202; F: T2-14438; G: T2-41011; H: T2-47003; I: T2-47331; J: T4-20296; K: T4-52638; L: T4-58706. Functional annotation of the unigenes (their homologs or similar genes) see Table 4. The *actin* gene was used as internal control. The number 1–16 indicate the sampling dates, and the details see S1 Table), that implied an important role in spike development. The functions of these genes should be investigated in detail.

### The molecular bases for abnormal spikes of D

Gene function loss can result in plant dwarf, such as an extreme dwarf phenotype of the GA-sensitive mutant of sunflower, *dwarf2* [80]. Pistillody in alloplasmic wheats is caused by alterations to the expression pattern of class-B MADS-box genes, *DL*, *AP1*, *AP2*, *FL* etc [29,52,53]. Rice hybrid sterility is the most common form of postzygotic reproductive isolation in plants. The hybrid sterility genes is in very different functional categories, aspartic protease, small ubiquitin-like modifier E3 ligase-like protein, F-box protein, transcription factor, the catalytic subunit of a Na^+^/K^+^ ATPase, polypeptide with a single MADF DNA-binding domain near its C terminus end [81]. These cases indicated that one gene mutation is sufficient to result in deleterious function and causing abnormal phenotype, even if a single amino acid exchange in the coding peptide. One phenotype may caused by different factors. Interestingly, the key genes involved in dwarfism, pistillody and sterility reported above were also important genes in wheat floral organ differentiation identified in this study, such as MADS-box, *DL*, *AP1*, *AP2*, *FL* [51, 66], E3 ligase-like protein, transcription factor [81], energy metabolism [79].

Although the genetic changes of mutant *dms* has not been identified yet, significant DEGs between T and D were carefully studied. The molecular base for abnormal spike differentiation of *dms* was proposed here (Fig 8). *TaFL* gene involves in specifying floral organ. A *YABBY* gene *TaDL* (DL) involves in specifying the pistil identity. The expressions of class A genes *TaAP1-2* and *TaAP2* were inhibited in D. The DNA replication was affected, most transcription factors were repressed, but some are activated. The biological processes of translation, photosynthesis, glycolysis, cell division are significantly repressed in D, signal transduction mechanism is activated. Furthermore, some unknown genes are suppressed or activated. These data constitute a frame work for transcriptome profile in D. Certainly it needs to be confirmed step by step in future.

## Conclusions

The wheat spike transcriptomes of D and T at early stage of floral organ differentiation were carefully analyzed. We identified 419 genes related to wheat spike differentiation, including typical homeotic genes, DNA replication, transcription and translation factors, carbohydrate transport and metabolism related genes. The identified homeotic genes were MADS-box (*MFO1*, *FUL2*, *FUL3*, *PAP2*, etc), *AP2*, *FL* and *DL*-like genes that played important roles in wheat floral organ differentiation. The expression levels of 143 DEGs in differentiating spikes were significantly different, the expression of *AP1*-like gene (*TaAP1-2*) was inhibited in D, and *AP2*-like gene (*TaAP2*) was significantly suppressed. Among the DEGs, 16.08% genes encoding transcription factors; 18.88% genes were of carbohydrate transport and metabolism, photosynthesis, energy production and conversion; more than 21% were novel and unknown function genes. Most biological processes such as transcription, translation, cell division, photosynthesis, carbohydrate transport and metabolism, energy production and conversion were repressed in D. Particularly most metabolism pathways of photosynthesis and carbohydrate metabolism were suppressed. Inhibition of homeotic genes, suppression of most biological processes and energy deficiency were considered as the major causal factors for abnormal spike development of D.

## Supporting Information

S1 Fig The proteins (marked in red) encoded by the highly expressed DEGs in the metabolism pathway of DNA replication. (Ko03030, Kanehisa laboratories).
(TIF)

S2 Fig The proteins (marked in red) encoded by the highly expressed DEGs in the spliceosome. (Ko03040, Kanehisa laboratories).
(TIF)

S3 Fig The proteins (marked in red) encoded by the highly expressed DEGs in the ribosome. (Ko03010, Kanehisa laboratories).
(TIF)

S1 Table Sampling dates and their corresponding spike developmental stages. (DOC)

S2 Table DNA sequences of the primers used in real-time QRT-PCR. (DOC)

S3 Table Functional classification of DEGs in young spikes between D (T2) and T (T4). (DOC)
