# Supplementary figures and images for: Transcriptome Analysis for Abnormal Spike Development of the Wheat Mutant dms
Source: PLoS One. 2016 Mar 16;11(3):e0149287. doi: 10.1371/journal.pone.0149287 (PMC4794226; doi:10.1371/journal.pone.0149287)

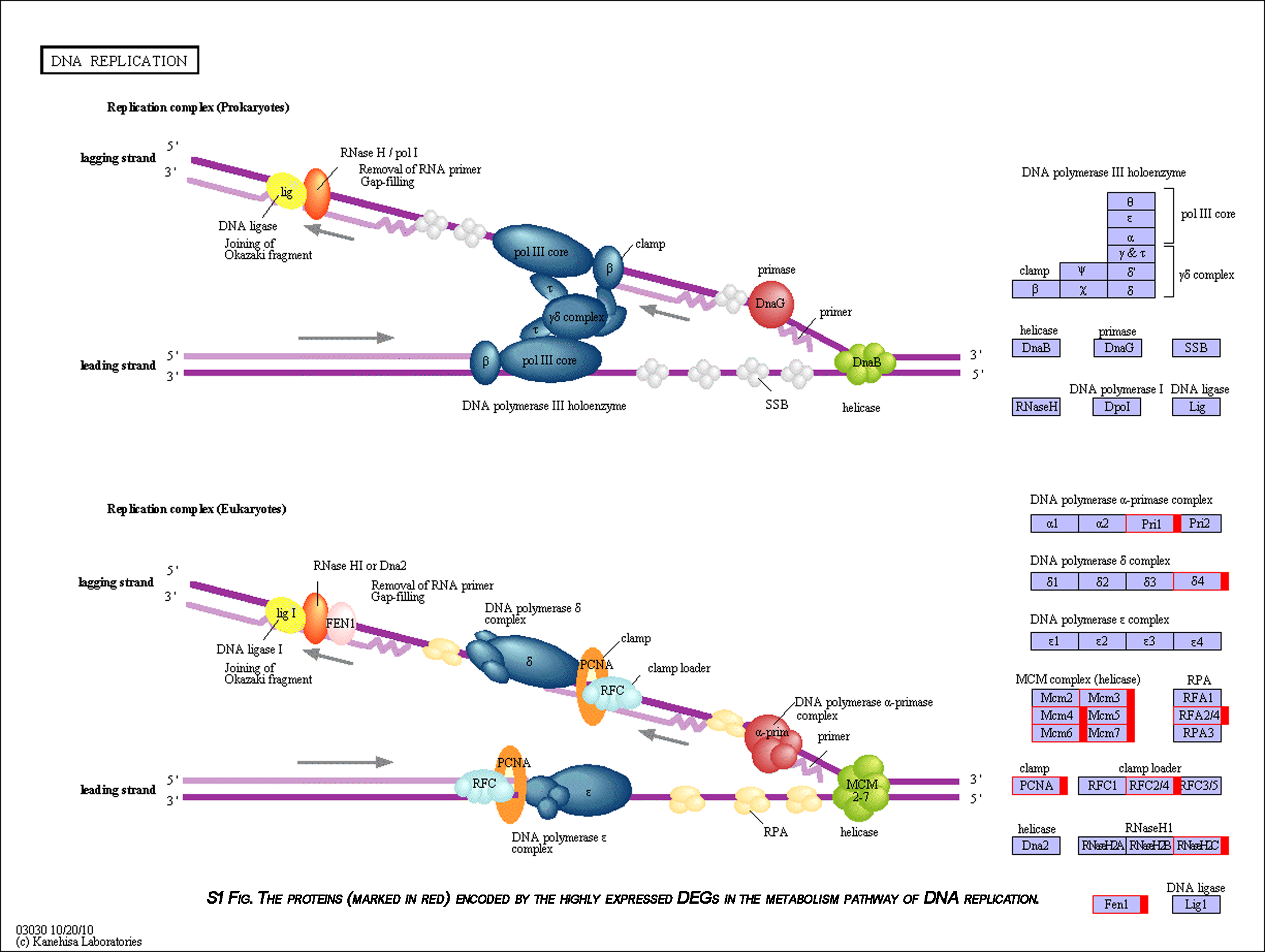

Supplement: S1 Fig — (Ko03030, Kanehisa laboratories). (TIF) [file pone.0149287.s001.tif]

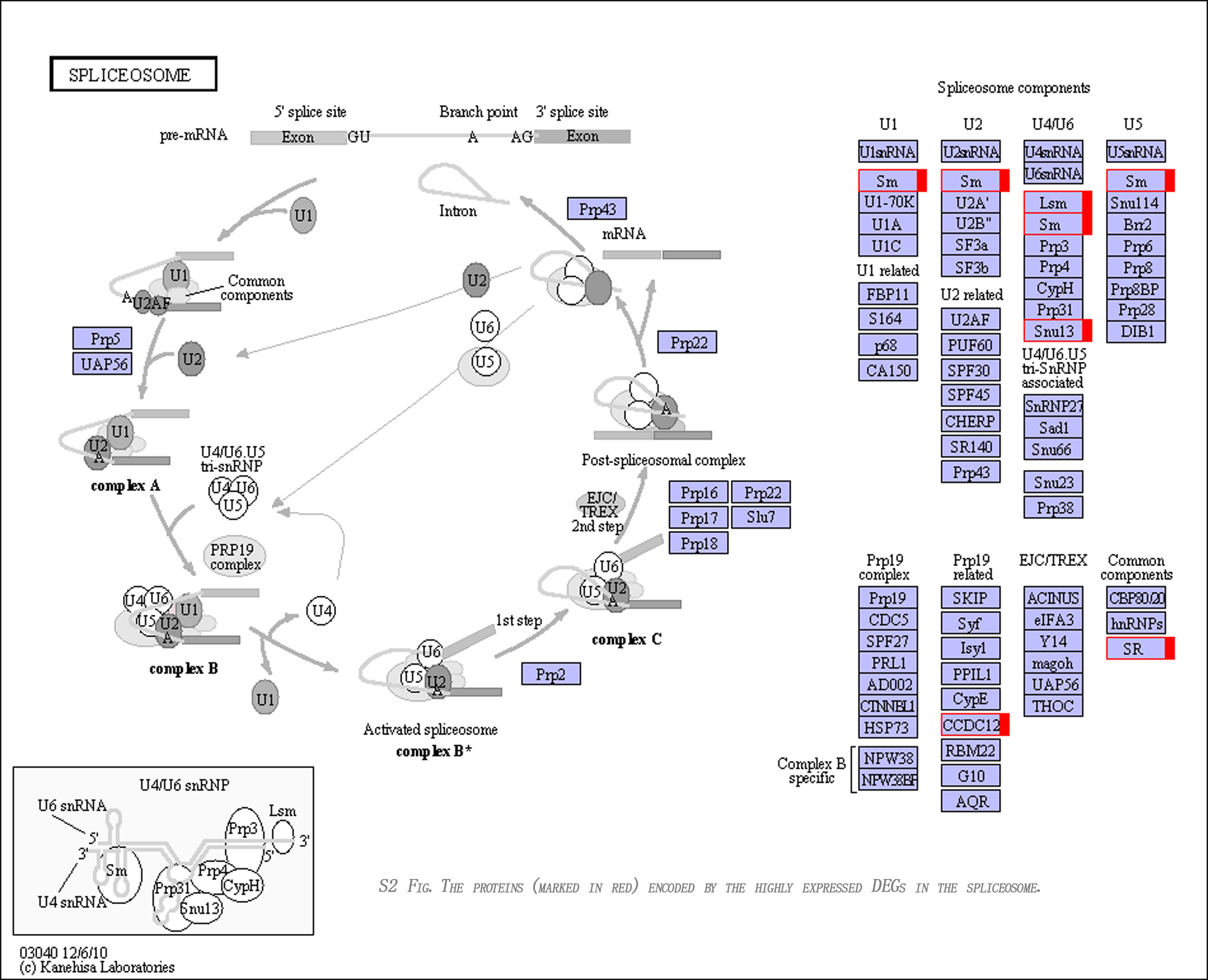

Supplement: S2 Fig — (Ko03040, Kanehisa laboratories). (TIF) [file pone.0149287.s002.tif]

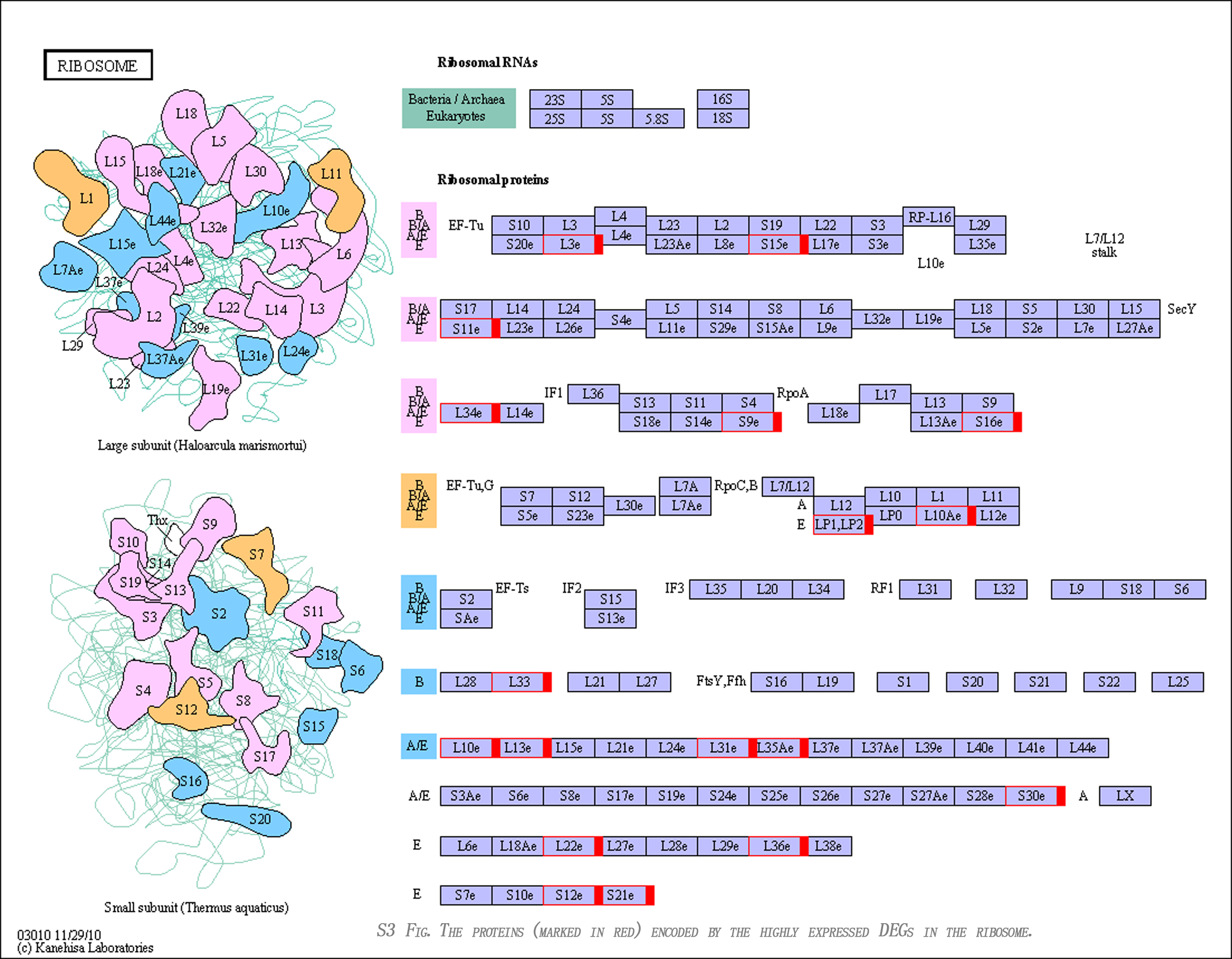

Supplement: S3 Fig — (Ko03010, Kanehisa laboratories). (TIF) [file pone.0149287.s003.tif]
