# Supplementary material for: Transcriptome Analysis for Abnormal Spike Development of the Wheat Mutant dms
Source: PLoS One. 2016 Mar 16;11(3):e0149287. doi: 10.1371/journal.pone.0149287 (PMC4794226; doi:10.1371/journal.pone.0149287)
Supplement: S1 Table — (DOC) [file pone.0149287.s004.doc]

**S1 Table. Sampling dates and their corresponding spike developmental stages.**

| **No.** | **Dates** | **A** | **B** | **C** | **D** | **E** | **F** | **G** | **H** | **I** | **J** | **K** | **L** | **M** | **N** | **O** | **P** | **Q** |
| --- | --- | --- | --- | --- | --- | --- | --- | --- | --- | --- | --- | --- | --- | --- | --- | --- | --- | --- |
| **1** | **1 March** | ★ | ▲ |  |  |  |  |  |  |  |  |  |  |  |  |  |  |  |
| **2** | **4 March** | ★ |  | ▲ |  |  |  |  |  |  |  |  |  |  |  |  |  |  |
| **3** | **8 March** | ★ |  |  | ▲ |  |  |  |  |  |  |  |  |  |  |  |  |  |
| **4** | **10 March** |  | ★ |  |  | ▲ |  |  |  |  |  |  |  |  |  |  |  |  |
| **5** | **12 March** |  |  | ★ |  |  | ▲ |  |  |  |  |  |  |  |  |  |  |  |
| **6** | **14 March** |  |  |  | ★ |  |  | ▲ |  |  |  |  |  |  |  |  |  |  |
| **7** | **16 March** |  |  |  |  | ★ |  |  | ▲ |  |  |  |  |  |  |  |  |  |
| **8** | **18 March** |  |  |  |  |  | ★ |  |  | ▲ |  |  |  |  |  |  |  |  |
| **9** | **20 March** |  |  |  |  |  |  | ★ |  |  | ▲ |  |  |  |  |  |  |  |
| **10** | **22 March** |  |  |  |  |  |  |  | ★ |  |  | ▲ |  |  |  |  |  |  |
| **11** | **25 March** |  |  |  |  |  |  |  |  | ★ |  |  | ▲ |  |  |  |  |  |
| **12** | **28 March** |  |  |  |  |  |  |  |  |  | ★ |  |  | ▲ |  |  |  |  |
| **13** | **31 March** |  |  |  |  |  |  |  |  |  |  | ★ |  |  | ▲ |  |  |  |
| **14** | **3 April** |  |  |  |  |  |  |  |  |  |  |  | ★ |  |  | ▲ |  |  |
| **15** | **7 April** |  |  |  |  |  |  |  |  |  |  |  |  | ★ |  |  | ▲ |  |
| **16** | **10 April** |  |  |  |  |  |  |  |  |  |  |  |  |  | ★ |  |  | ▲ |

▲: Zhoumai 18, T and M share the same development rate.

★: The development rate of D is slower than that of Zhoumai 18, T and M.

A: pre-double ridge stage

B: early double ridge stage

C: metaphase of double ridge

D: anaphase of double ridge

E: glume differentiation stage

F: early stage of floret development

G: metaphase of floret development

H: anaphase of floret development

I: early stage of stamen / pistil differentiation

J: metaphase of stamen / pistil differentiation

K: anaphase of stamen / pistil differentiation

L: early stage of carpel differentiation

M: anaphase of carpel differentiation

N: stigma primodium differentiation

O: style elongation stage

P: stigma formation stage

Q: stigma elongation stage

The developmental stages were recorded according to the report of Cui et al. (2008)
