# Supplementary material for: Transcriptome Analysis for Abnormal Spike Development of the Wheat Mutant dms
Source: PLoS One. 2016 Mar 16;11(3):e0149287. doi: 10.1371/journal.pone.0149287 (PMC4794226; doi:10.1371/journal.pone.0149287)
Supplement: S2 Table — (DOC) [file pone.0149287.s005.doc]

**S2 Table. DNA sequences of the primers used in real-time QRT-PCR.**

| **Unigene ID** | **GeneBank Accession** | **Forward primer (5′-3′)** | **Backward primer (5′-3′)** | **Product length (bp)** | **Tm**  **(^o^C)** |
| --- | --- | --- | --- | --- | --- |
| **T4-58706** |  | ATGAGGACTTGCCAGAGATGAATGC | CTCACGAACAGGTGCTGGAACAAT | 113 | 55.3 |
| **T4-52638** |  | ACGGCACGACTCCTGTATCCTT | GATGTTGCTGGTCTGTGGTACTTCA | 103 | 55.6 |
| **T2-14438** |  | CTTGAGCGGCATTCTGATGGAAGA | ATTGGATGCGGTAGGTCAGTCTGT | 181 | 57.7 |
| **T2-47331** |  | CTGAGGAGGACGCCTGACAATAATC | GCGAATGAGACCGATGTGAAGTGT | 205 | 56.3 |
| **T2-41011** |  | AGATGCTCCGAACCTGCCATATCA | CTACGCCGTCTGTCCTTCCTCTTA | 114 | 57.7 |
| **T2-47003** |  | ACCTACAAGACCGTGACCTGATGTT | AGGTGATGTCCTCCTTGGCTGATAG | 204 | 56.9 |
| **T1-56016** |  | GACGACGCCACAGGAATCACAT | GCCGTCAGGTAGAATCGTAGAACAG | 201 | 58.3 |
| **T1-71202** |  | CGGCTGATTCTCCTGGACTACGAT | CGCACAGACGATTCAAGACAGAGAT | 100 | 56.9 |
| **T1-49256** |  | TTGCTCACATGCGGATTCCTCTG | CACACTCACTCGTTGTCATCACTCA | 76 | 52.3 |
| **T1-47968** |  | TCGCTCAGGTTCCTCGTGTTCAA | TCCACACCATTCAACGCATTCAGAG | 202 | 58.0 |
| **T1-38135** |  | GGCTCTTGTAGAAGGCAGCATACTC | GGAGGGCAAGGTTGAGGAAATTGAT | 170 | 55.9 |
| **T4-20296** |  | TTGCCTCATCGGTCTCGCTCTAT | GGCTGTTCCTTGGTGAAGAATTTGG | 95 | 53.2 |
| ***Actin*** | gi:48927617 | CCAAGGCGGAGTACGATGAGTCT | TTCATACAGCAGGCAAGCACCAT | 246 | 56.5 |
| ***GAPDH*** | gi:7579063 | ACTAACTGCCTTGCTCCTCTTGCTA | CCAGTGCTGCTTGGAATGATGTTGA | 176 | 56.8 |

The transcript of *actin* or *gadph* gene was chosen as internal controls for data normalization.

*GADPH:* glyceraldehyde-3-phosphate dehydrogenase.
