# Supplementary material for: Transcriptome Analysis for Abnormal Spike Development of the Wheat Mutant dms
Source: PLoS One. 2016 Mar 16;11(3):e0149287. doi: 10.1371/journal.pone.0149287 (PMC4794226; doi:10.1371/journal.pone.0149287)
Supplement: S3 Table — (DOC) [file pone.0149287.s006.doc]

**S3 Table. Functional classification of DEGs in young spikes between D (T2) and T (T4).**

| **Functional class** | **DEG Id** | **Log_2_(T4/T2)**  **(T4:T2)** | **Gene ID* and annotation**  **(Swissprot, TrEMBL, NT or NR)** | **Functional annotation**  **(GO)** |
| --- | --- | --- | --- | --- |
| **Cellular component** | T4-64989 | 2.81  (35:5) | tr|M0WMB0|; M0WMB0_HORVD, Glycoprotein gp2 | cellular component organization; nuclear lumen |
|  | T3-2528 | 1.01  (97:48) | sp|O65351|; Xylem serine proteinase 1; Subtilisin-like protease | serine-type endopeptidase activity; cell wall; seed coat development |
|  | T3-19149 | 14.92  (31:0) | tr|Q2QRM1|; 1Ds prolamin gene locus; Retrotransposon protein, putative, Ty1-copia subclass | prolamin |
|  | T2-53412 | -2.49  (10:56) | sp|Q6K602|; Bidirectional sugar transporter SWEET15; Protein RUPTURED POLLEN GRAIN 1 | protein binding; pollen development; anther dehiscence; cellular response to osmotic stress |
|  | T3-4632 | -2.54  (5:29) | sp|Q10LN5|; Bidirectional sugar transporter SWEET16; Protein RUPTURED POLLEN GRAIN 1 | integral to plasma membrane; cellular response to osmotic stress |
|  | T1-55320 | -4.46  (1:22) | tr|M0V076|; hypothetical protein TRIUR3_31666 | cytoplasmic membrane-bounded vesicle |
|  | T2-46634 | -1.64  (24:75) | sp|Q9C5S0|; Classical arabinogalactan protein 9 | Binding; multicellular organismal development; anatomical structure morphogenesis |
|  | T1-46664 | -1.99  (46:183) | sp|Q8T4N5|; Proline and lysine rich protein | hydrolase activity; virus-host interaction; cytoplasmic membrane-bounded vesicle |
|  | T4-20296 | 2.35  (56:11) | sp|Q652A8|; UDP-glucose 4-epimerase 3 | UDP-glucose 4-epimerase activity; pollen development; root epidermal cell differentiation |
|  | T2-39457 | -3.70  (3:39) | sp|Q08000|; Em protein H2; Em-D1 | Cytoplasm; regulation of flower development; meristem structural organization; vegetative to reproductive phase transition of meristem |
| **Energy production and conversion** | T4-34310 | 2.41  (155:29) | sp|Q9SLZ0|; Phosphoenolpyruvate carboxykinase [ATP] | DNA binding; phosphoenolpyruvate carboxykinase (ATP) activity |
|  | T4-16243 | 3.49  (45:4) | sp|Q02979|; Glycerophosphodiester phosphodiesterase GDE1 | glycerol metabolic process; |
|  | T1-65690 | 1.34  (576:227) | sp|P05336|; alcohol dehydrogenase ADH1A | alcohol dehydrogenase (NAD) activity |
|  | T3-1839 | 1.15  (138:62) | sp|P22988|; L-lactate dehydrogenase A | L-lactate dehydrogenase activity |
|  | T3-53572 | 4.52  (23:1) | sp|Q6K548|; Mitochondrial outer membrane protein porin 1 | Transport; membrane |
|  | T3-5246 | 1.83  (203:57) | sp|Q5E9D6|; Uncharacterized protein; Haloacid dehalogenase-like hydrolase domain-containing protein 3 | Mitochondrion; phosphoglycolate phosphatase activity |
|  | T3-37606 | 1.96  (117:30) | sp|A2YIW7|; Thioredoxin H-type | enzyme inhibitor activity; cell wall |
| **Replication, recombination and repair** | T1-42449 | -4.86  (1:29) | sp|P30022|; Tegument protein VP22 | Replication, recombination and repair |
|  | T3-64459 | -4.70  (1:26) | sp|P0C230|; Protamine; | DNA metabolic process; calcium ion binding; transcription from RNA polymerase II promoter; mRNA processing |
|  | T4-58706 | 4.58  (24:1) | sp|Q92372|; Replication factor A protein 1 | Replication, recombination and repair |
| **Transcription** | T2-16245 | 14.36  (21:0) | sp|Q6ZQP7|; Uncharacterized protein LOC284861 | Binding; RNA processing; transport; nuclear part |
|  | T3-2855 | 3.09  (34:4) | sp|Q55750|; Transcription-repair-coupling factor | damaged DNA binding; helicase activity |
|  | T2-54631 | 1.51  (91:32) | sp|Q9FUD3|; Basic leucine zipper 9; Regulatory protein opaque-2 | sequence-specific DNA binding transcription factor activity; regulation of transcription, DNA-dependent; positive regulation of seed maturation |
|  | T3-5762 | 1.28  (68:28) | sp|Q9FUD3|; Basic leucine zipper 9; Regulatory protein opaque-2 | sequence-specific DNA binding transcription factor activity; positive regulation of seed maturation |
|  | T3-63268 | 2.29  (38:9) | sp|Q8IYB3|; Serine/arginine repetitive matrix protein 1 | RNA processing; nuclear part |
|  | T4-16626 | 2.93  (190:25) | sp|Q8IYB3|; Serine/arginine repetitive matrix protein 1 | RNA processing; nuclear part |
|  | T2-39084 | 2.91  (196:26) | sp|Q8IYB3|; Serine/arginine repetitive matrix protein 1 | RNA processing; nuclear part |
|  | T2-47331 | -4.43  (2:43) | tr|F2DQE5|; Serine/arginine repetitive matrix protein 1 |  |
|  | T2-47003 | 1.84  (86:24) | sp|Q94BN0|; BTB/POZ and TAZ domain-containing protein 2 | transcription cofactor activity; response to cold; pollen development; system development |
|  | T2-31732 | 1.21  (88:38) | sp|Q6ZQP7|; Uncharacterized protein LOC284861 | Binding; Nucleoplasm; RNA processing |
|  | T2-43621 | -1.45  (30:82) | sp|Q8VYA5|; Serine/arginine-rich splicing factor RS2Z33; Splicing factor, arginine/serine-rich 4 | nucleic acid binding; mRNA splicing, via spliceosome; regulation of transcription, DNA-dependent |
|  | T4-42005 | -3.58  (3:36) | sp|Q9T0K5|; Leucine-rich repeat extensin-like protein 3 | mRNA splicing, via spliceosome; developmental process involved in reproduction |
|  | T1-64651 | -3.09  (6:51) | sp|Q10QF2|; Homeobox-leucine zipper protein HOX12 | transcription regulatory region sequence-specific DNA binding; root development |
| **Translation, ribosomal structure and biogenesis** | T4-34116 | 2.61  (128:21) | sp|Q42202|; Ubiquitin-60S ribosomal protein L40-2 | structural constituent of ribosome |
|  | T3-67771 | 1.21  (102:44) | sp|Q0JGY1|; 60S ribosomal protein L5-1 | nuclear-transcribed mRNA catabolic process, nonsense-mediated decay; leaf morphogenesis |
|  | T3-67769 | 1.04  (396:193) | sp|Q0JGY1|; 60S ribosomal protein L5-1 | structural constituent of ribosome |
|  | T3-64098 | 1.06  (205:98) | sp|Q41852|; 40S ribosomal protein S21 | structural constituent of ribosome; |
|  | T4-52781 | 2.49  (56:10) | sp|Q2IPZ7|; Translation initiation factor IF-2 | catalytic activity |
|  | T3-59805 | 1.03  (695:341) | sp|P13728|; Salivary glue protein Sgs-3; Triticum aestivum cold acclimation protein WCOR825 | DNA binding; transcription, DNA-dependent; ribonucleoprotein complex |
|  | T3-10313 | 1.21  (88:38) | sp|Q945F4|; Eukaryotic translation initiation factor 5A-2 | translation initiation factor activity; xylem development |
|  | T4-61340 | 1.07  (130:62) | sp|Q9FN11|; LOB domain-containing protein 37 | regulation of transcription, DNA-dependent; simple leaf morphogenesis |
|  | T4-61565 | -1.02  (130:264) | sp|A8M746|; Translation initiation factor IF-2 | Binding; nuclear part |
|  | T2-41011 | -14.49  (0:23) | sp|Q9FR37|; Amidase 1 | amidase activity; indoleacetic acid biosynthetic process; leaf morphogenesis; cell differentiation |
|  | T1-64019 | -3.52  (2:23) | sp|Q9FR37|; Amidase 1 | amidase activity; leaf morphogenesis; cell differentiation |
| **Posttranslational modification, protein turnover, chaperones** | T1-38135 | 1.38  (135:52) | sp|Q0J4P2|; Heat shock protein 81-1 | ATP binding; protein folding; flower development; negative regulation of seed germination; leaf development |
|  | T3-57349 | -1.34  (83:210) | sp|Q9LHA8|; Probable mediator of RNA polymerase II transcription subunit 37c | protease binding; receptor activity |
|  | T4-55787 | -1.11  (95:205) | sp|P27484|; Glycine-rich protein 2 | RNA binding transcription antitermination factor activity (GO:0001072); stamen development |
|  | T1-59183 | -2.08  (9:38) | sp|Q5Z5B2|; Protein argonaute 1D | RNA 7-methylguanosine cap binding; embryonic pattern specification; polarity specification of adaxial/abaxial axis; leaf morphogenesis; primary shoot apical meristem specification; maintenance of shoot apical meristem identity; somatic stem cell maintenance; flower morphogenesis |
| **Amino acid transport and metabolism** | T1-65055 | 1.37  (75:29) | sp|P52894|; Alanine aminotransferase 2 | gamma-glutamyltransferase activity |
|  | T3-2970 | 2.10  (86:20) | sp|Q8GT75|; NEP1-interacting protein 1 | ubiquitin-protein ligase activity; amino acid transport; stamen development |
|  | T2-61231 | 1.01  (99:49) | sp|Q38946|; Glutamate dehydrogenase 2 | glutamate dehydrogenase (NAD+) activity |
|  | T3-50080 | 1.03  (92:45) | sp|P31862|; Bowman-Birk type wound-induced proteinase inhibitor WIP1 | serine-type endopeptidase inhibitor activity; proteolysis |
|  | T1-65680 | -1.23  (49:115) | sp|P31752|; Asparagine synthetase [glutamine-hydrolyzing]; glutamine-dependent asparagine synthetase | asparagine synthase (glutamine-hydrolyzing) activity; |
|  | T4-60834 | -1.08  (258:545) | sp|Q10QA5|; Probable strigolactone esterase D14 | carboxylesterase activity; cellular protein metabolic process |
|  | T3-66792 | -1.08  (117:373) | sp|Q10QA5|; Probable strigolactone esterase D14 | carboxylesterase activity; plastid; intracellular organelle lumen |
|  | T2-50731 | -14.87  (0:30) | sp|P31862|; Bowman-Birk type wound-induced proteinase inhibitor WIP1; wali5 | serine-type endopeptidase inhibitor activity; peptidase activity; proteolysis |
|  | T4-20804 | -2.77  (5:34) | sp|P09864|; Bowman-Birk type proteinase inhibitor II-4 | serine-type endopeptidase inhibitor activity; negative regulation of peptidase activity |
| **Signal transduction mechanisms** | T1-59554 | -2.98  (16:126) | sp|Q9SPK4|; Phosphoenolpyruvate carboxylase kinase 1；chromosome 1Ds prolamin gene locus | nucleotide binding; protein phosphorylation; regulation of pollen tube growth |
|  | T3-13958 | -1.62  (39:120) | sp|Q39030|; Serine/threonine-protein kinase AtPK2/AtPK19 | positive regulation of cell differentiation; protein kinase B signaling cascade; negative regulation of cell proliferation; positive regulation of cell growth |
|  | T2-52632 | -1.42  (24:64) | sp|Q84JI0|; Probable protein phosphatase 2C 30 | protein serine/threonine phosphatase activity |
|  | T1-52792 | -2.25  (470:2240) | sp|Q05349|; Auxin-repressed 12.5 kDa protein | response to sucrose stimulus |
|  | T1-56016 | -5.17  (1:36) | sp|A2X7U1|; Homeobox-leucine zipper protein HOX24 | transcription regulatory region sequence-specific DNA binding; regulation of transcription, DNA-dependent |
| **Response to out stimulus** | T3-7355 | 1.25  (204:86) | sp|P0C5C6|; Protein GOS9; jasmonate-induced protein | glucose binding; mannose binding; response to jasmonic acid stimulus; |
|  | T1-49755 | 1.77  (65:19) | sp|Q8LGG8|; Universal stress protein A-like protein; | response to stress |
|  | T4-44627 | 2  (128:32) | sp|P26154|; Cold-regulated protein BLT14; Pm3b-like disease resistance protein | defense response |
|  | T4-722 | 4.70  (26:1) | sp|Q9S740|; Lysine-rich arabinogalactan protein 19; Pathogenesis-related protein 1-like protein | defense response |
|  | T3-34007 | 3.84  (43:3) | tr|L0AR00|; Salt-induced protein GN=SAIN2 | response to salt stress; response to stress |
|  | T2-46647 | 1.35  (183:72) | sp|P42736|; Ethylene-responsive transcription factor RAP2-3; AP2/EREBP-like protein | respiratory burst involved in defense response; organ senescence |
|  | T1-48485 | 2.04  (78:19) | sp|P46518|; Late embryogenesis abundant protein Lea14-A | protein binding; response to desiccation |
|  | T3-67901 | 1.87  (51:14) | sp|P46519|; Desiccation protectant protein Lea14 homolog; Late embryogenesis abundant protein Lea14-A | response to desiccation; embryo development ending in seed dormancy |
|  | T4-918 | 4.70  (26:1) | sp|P14928|; cold acclimation protein WCOR615; ABA-inducible protein PHV A1 | RNA processing; nuclear lumen; organic cyclic compound binding |
|  | T3-993 | 1.31  (208:84) | sp|P46524|; cold acclimation protein WCOR410c; Dehydrin COR410 | nucleic acid binding; response to stress; response to temperature stimulus; regulation of biological process |
|  | T2-48570 | 1.23  (422:180) | sp|Q939N5|; Platelet binding protein GspB | protein binding; response to stress |
|  | T4-59999 | 3.64  (25:2) | sp|Q939N5|; Platelet binding protein GspB | protein binding; cellular process |
|  | T3-66375 | -1.32  (43:107) | sp|Q948Z4|; Snakin-1; gibberellin stimulated transcript (GAST1) | cell proliferation; regulation of reactive oxygen species metabolic process |
|  | T2-42967 | -1.40  (33:87) | sp|Q948Z4|; Snakin-1; gibberellin stimulated transcript | protein binding; plasma membrane; cell proliferation |
| **Cell cycle control, cell division, chromosome partitioning** | T4-52057 | 2.40  (237:45) | sp|Q02921|; Early nodulin-93; Early nodulin protein GN=57h21.33 | nodulation; cellular protein modification process |
|  | T1-59713 | 2.46  (269:49) | sp|Q02921|; Early nodulin-93 | integral to membrane; |
|  | T1-53924 | 1.15  (853:385) | sp|Q9FPQ6|; Vegetative cell wall protein gp1 ;Predicted protein | nucleic acid binding;protein binding |
|  | T1-47968 | 1.22  (112:48) | sp|O02414|; Dynein light chain LC6, flagellar outer arm | establishment of mitotic spindle orientation; pollen tube growth |
|  | T4-51695 | 1.27  (137:57) | sp|O02414|; Dynein light chain LC6, flagellar outer arm | pollen tube growth; regulation of cell proliferation |
|  | T2-40114 | 1.29  (164:67) | sp|Q759T0|; Dynein light chain 1; Dynein light chain LC6, flagellar outer arm | establishment of mitotic spindle orientation; root hair cell development |
| **Photosynthesis** | T4-60933 | 1.01  (246:122) | sp|P36886|; Photosystem I reaction center subunit psaK, chloroplastic | Photosynthesis; photosystem I |
|  | T3-13133 | 1.31  (72:29) | sp|Q10HD0|; Chlorophyll a-b binding protein, chloroplastic | photosynthesis, light harvesting; photosystem I; photosystem II |
|  | T3-586 | 1.30  (326:132) | sp|P27525|; Chlorophyll a-b binding protein CP24 10B; 10A | photosystem II antenna complex; light harvesting; |
|  | T2-40779 | 1.26  (204:85) | sp|Q00434|; Oxygen-evolving enhancer protein 2, chloroplastic | chloroplast thylakoid membrane; subunit of oxygen evolving system of photosystem II |
|  | T2-48466 | 1.03  (333:163) | sp|P27523|; Chlorophyll a-b binding protein of LHCII type III, chloroplastic | photosystem II; photosynthesis, light harvesting |
|  | T3-66750 | 1.36  (349:136) | sp|Q0D5P8|; Oxygen-evolving enhancer protein 3, chloroplastic | chloroplast thylakoid membrane; photosystem II assembly; photosynthesis |
|  | T3-68563 | 1.24  (180:76) | sp|Q07473|; Chlorophyll a-b binding protein CP29.2, chloroplastic | thylakoid light-harvesting complex |
|  | T3-8604 | 1.84  (86:24) | sp|Q6Z4A7|; adenylylsulfate reductase 1, chloroplastic | phosphoadenylyl-sulfate reductase (thioredoxin) activity; chloroplast thylakoid |
|  | T3-5912 | 1.31  (67:27) | sp|P09315|; Glyceraldehyde-3-phosphate dehydrogenase A, chloroplastic | glyceraldehyde-3-phosphate dehydrogenase (NAD+) (phosphorylating) activity; photosynthetic electron transport in photosystem I |
|  | T3-363 | 2.40  (58:11) | sp|Q8LJJ9|; Acyl-[acyl-carrier-protein] desaturase 1, chloroplastic | stearoyl-CoA 9-desaturase activity; sulfur amino acid metabolic process; unsaturated fatty acid biosynthetic process; chloroplast |
| **Carbohydrate transport and metabolism** | T4-52638 | 14.14  (18:0) | sp|Q7XUR3|; Putative alpha-L-fucosidase 1 | monooxygenase activity; oxidation-reduction process |
|  | T4-55706 | 2.08  (59:14) | sp|Q8RX87|; Probable galactinol--sucrose galactosyltransferase 6 | alpha-galactosidase activity; sucrose biosynthetic process |
|  | T4-33351 | 1.27  (264:109) | sp|Q94AA4|; 6-phosphofructokinase 3 | 6-phosphofructokinase activity; fructose 6-phosphate metabolic process |
|  | T3-4607 | 1.25  (179:75) | sp|Q94AA4|; 6-phosphofructokinase 3 | 6-phosphofructokinase activity |
|  | T4-57047 | 3.00  (48:6) | sp|Q9SDL8|; Fructose-1,6-bisphosphatase, cytosolic | fructose 1,6-bisphosphate 1-phosphatase activity; carbohydrate metabolic process; negative regulation of cell growth |
|  | T3-5013 | 2.19  (164:36) | sp|Q2UXF7|; Fructan 6-exohydrolase | sucrose alpha-glucosidase activity; cell wall |
|  | T3-10446 | 1.13  (321:147) | sp|A2XFI3|; Pyruvate decarboxylase isozyme 2 | pyruvate decarboxylase activity |
|  | T3-840 | 2.46  (226:41) | sp|Q5Z8T3|; Probable inositol oxygenase | inositol oxygenase activity; inositol catabolic process; inositol phosphate-mediated signaling |
|  | T4-33846 | 1.78  (55:16) | sp|Q9LUV2|; Probable protein Pop3 | Glycolysis; hyperosmotic response; chlorophyll biosynthetic process |
|  | T1-71202 | -1.01  (76:153) | sp|Q0WUI9|; trehalose-6-phosphate synthase 2 | alpha,alpha-trehalose-phosphate synthase (UDP-forming) activity; specification of floral organ identity; carpel development; meristem development |
| **Plastid** | T2-29673 | -5.71  (2:72) | sp|Q9UQ35|; Serine/arginine repetitive matrix protein 2; Salt tolerant correlative protein | Binding; Plastid; nuclear lumen |
|  | T2-40540 | -3.09  (4:34) | tr|M0YZ23|; hypothetical protein | Mitochondrion; plastid |
|  | T1-65222 | -3.75  (2:27) | sp|P43396|; Metallothionein-like protein 1; metallothionein | copper ion binding; response to osmotic stress; plastid |
|  | T3-14820 | 2.48  (28:5) | sp|Q9UF83|; Uncharacterized protein DKFZp434B061 | plastid |
| **lipid transport and metabolism** | T4-60861 | -1.04  (364:750) | sp|O65091|; Non-specific lipid-transfer protein 4 | lipid binding; lipid transport; response to wounding |
|  | T1-56152 | -5.13  (1:35) | sp|Q9LY00|; Probable WRKY transcription factor 70; WRKY45 transcription factor | sequence-specific DNA binding transcription factor activity; MAPK cascade; immune effector process; systemic acquired resistance |
|  | T4-16051 | -1.24  (41:97) | sp|Q6L545|; Gibberellin receptor GID1 | protein binding; MAPK cascade; gibberellic acid mediated signaling pathway |
|  | T2-50516 | 2.75  (47:7) | tr|M0YYN8|; predicted protein | glycerophosphodiester phosphodiesterase activity; glycerol metabolic process |
| **Inorganic ion transport and metabolism** | T2-43231 | -3.27  (3:29) | sp|Q67YC0|; Inorganic pyrophosphatase 1 | inorganic diphosphatase activity; organ development; negative regulation of transcription, DNA-dependent |
| **General function** | T3-364 | 2.41  (53:10) | sp|Q96PM5|; RING finger and CHY zinc finger domain-containing protein 1; CHY-type zinc finger protein | ubiquitin ligase complex; protein autoubiquitination |
|  | T1-49256 | -5.36  (1:41) | sp|P20186|; Uncharacterized 35.5 kDa protein in transposon Tn4556; LEA protein 12 (LEA12) | alcohol dehydrogenase (NAD) activity; response to salt stress; response to hypoxia; response to stress |
|  | T1-68518 | -3.64  (2:25) | sp|P55308|; Catalase isozyme 2 | catalase activity; glyoxysome; oxidation-reduction process |
|  | T1-67451 | -4.32  (1:20) | sp|P93147|; Isoflavone 2’-hydroxylase | monooxygenase activity; heme binding; electron transport chain |
|  | T2-35350 | -3.73  (11:146) | sp|Q42980|; Oleosin 16 kDa | mRNA export from nucleus; meristem structural organization; vegetative to reproductive phase transition of meristem; regulation of flower development |
|  | T4-55876 | 1.36  (386:150) | sp|Q8BTI8|; Serine/arginine repetitive matrix protein 2 | protein binding; vegetative phase change; cellular transition metal ion homeostasis |
| **Unkown** | T4-2553 | 14.21  (19:0) | tr|F2DSD0|; Uncharacterized protein；splicing factor 3A subunit 1-like |  |
|  | T2-33556 | 5.43  (43:1) | gi|474188572|; hypothetical protein; Setaria italica titin-like |  |
|  | T4-61200 | 2.77  (82:12) | tr|F2D0R8|; hypothetical protein |  |
|  | T2-36225 | 1.22  (77:33) | tr|F2DMA8|; Predicted protein |  |
|  | T1-31419 | -1.26  (82:197) | tr|F2EL72|; Predicted protein |  |
|  | T4-15960 | -1.16  (46:103) | tr|C7IYE4|; Os02g0557000 protein (Fragment) |  |
|  | T1-31712 | 1.35  (102:40) | gi|473886408|; hypothetical protein |  |
|  | T1-64318 | 3.00  (55:7) | tr|M0W9L9|; Uncharacterized protein LOC284861 |  |
|  | T2-14438 | 4.86  (29:1) | tr|R7W5L8|; hypothetical protein F775_20975 (*A. tauschii*) |  |
|  | T4-61132 | 1.97  (157:40) | tr|M0YGX0|; Uncharacterized protein LOC284861 |  |
|  | T1-41681 | -3.46  (2:22) | tr|M0Y9G9|; Uncharacterized protein |  |
|  | T3-33825 | 4.32  (20:1) | tr|F2CYY9|; Predicted protein | 3B snp |
|  | T3-60830 | 2.09  (64:15) | gi|473999163|; hypothetical protein |  |
|  | T3-51735 | 2.67  (51:8) | tr|F2D0R8|; Predicted protein |  |
|  | T4-63074 | 13.97  (16:0) | gi|474299676|; hypothetical protein；G-protein coupled receptor 114-like |  |
|  | T3-1172 | -1.12  (74:161) | tr|F2CQP4|; Auxin-repressed 12.5 kDa protein |  |
|  | T3-44293 | 3.08  (68:8) | dbj|AK334784.1|; Leucine-rich repeat extensin-like protein 5 |  |
|  | T4-60716 | 1.55  (91:31) | gb|U49104.1|; calmodulin TaCaM3-3 |  |
|  | T4-16532 | 2.69  (71:11) | tr|F2DV27|; Uncharacterized protein |  |
|  | T1-44629 | -4.52  (1:23) | tr|I1H6P4|; Uncharacterized protein | mitochondrion |
|  | T2-63525 | -1.84  (45:161) | tr|F2E9M7|; Predicted protein |  |
|  | T4-56204 | 3.42  (32:3) | tr|M0Z490|; Uncharacterized protein；wound induced protein mRNA |  |
|  | T4-20316 | 2.81  (35:5) | tr|M0ZBN5|; Formin-like protein 7 |  |
|  | T2-41471 | -3.50  (3:34) | tr|F2CUD0|; Predicted protein; TaIPS1.1 miRNA gene；Black Hulless IPS1 mRNA |  |
|  | T3-62824 | 1.40  (66:25) | tr|F2D2M5|; Keratin-associated protein 10-11 |  |
|  | T3-10221 | 2.29  (39:8) | tr|M0YLV5|; 2-aminoethanethiol dioxygenase |  |

* The accession number with ‘sp’ is that of database SwissProt, with ‘tr’ is that of TrEMBL, with ‘gb’ is that of NT, with ‘gi’ is that of NR database.
